# Architecture of the outer-membrane core complex from a conjugative type IV secretion system

**DOI:** 10.1038/s41467-021-27178-8

**Published:** 2021-11-25

**Authors:** Himani Amin, Aravindan Ilangovan, Tiago R. D. Costa

**Affiliations:** 1 MRC Centre for Molecular Bacteriology and Infection, Department of Life Sciences, Imperial College, London, SW7 2AZ UK; 2 School of Biological and Chemical Sciences, Queen Mary University of London, London, E1 4NS UK

**Keywords:** Membrane proteins, Bacterial secretion, Cryoelectron microscopy

## Abstract

Conjugation is one of the most important processes that bacteria utilize to spread antibiotic resistance genes among bacterial populations. Interbacterial DNA transfer requires a large double membrane-spanning nanomachine called the type 4 secretion system (T4SS) made up of the inner-membrane complex (IMC), the outer-membrane core complex (OMCC) and the conjugative pilus. The iconic F plasmid-encoded T4SS has been central in understanding conjugation for several decades, however atomic details of its structure are not known. Here, we report the structure of a complete conjugative OMCC encoded by the pED208 plasmid from *E. coli*, solved by cryo-electron microscopy at 3.3 Å resolution. This 2.1 MDa complex has a unique arrangement with two radial concentric rings, each having a different symmetry eventually contributing to remarkable differences in protein stoichiometry and flexibility in comparison to other OMCCs. Our structure suggests that F-OMCC is a highly dynamic complex, with implications for pilus extension and retraction during conjugation.

## Abstract (web summary)

DNA transfer between two bacterial cells is mediated by the conjugative type 4 secretion systems (T4SSs). Here, the authors report the structure of a complete T4SS outer-membrane core complex (OMCC), revealing distinct C17 and C13 symmetries of its central inner and peripheral outer ring regions, respectively.

## Introduction

First described in the 1940s by Lederberg and Tatum, conjugation is a remarkable phenomenon whereby single-stranded DNA is delivered unidirectionally from a donor to recipient cell in bacteria^1^. As an elaborate form of horizontal gene transfer, this process is crucial for bacterial evolution and influences genome plasticity. Concerningly, it is a major cause for the widespread transmission and persistence of antibiotic resistance amongst bacterial populations^2–4^.

The conjugative transfer is mediated by a type IV secretion system (T4SS); a specialised membrane-embedded secretion machine found in Gram-negative and Gram-positive bacteria^5^. T4SSs are incredibly versatile nanomachines capable of secreting transforming DNA into plant cells^6^ and lethal protein effectors to competing bacteria and eukaryotic cells during infection^7–11^.

Minimal conjugative machineries in Gram-negative bacteria are composed of 12 protein subunits. This includes the archetypal VirB/D system from *Agrobacterium tumefaciens*, where the T4SS is composed of the proteins VirB1-11 and VirD4, which are organised into an outer membrane core complex (OMCC), an inner-membrane complex (IMC) and an extracellular pilus^12–17^. The canonical OMCC adopts a barrel-shaped architecture consisted of two ringed layers, the outer (O) and inner (I) layers. The overall structure is composed by 14 copies of the VirB7, VirB9 and VirB10 proteins^18–20^. VirB10 proteins span both the inner- (IM) and outer-membranes (OM) via their N-terminal domains (NTD) and the two-helix bundle located at the C-terminal domain (CTD) of the proteins^19,21^. The VirB7 lipoproteins are covalently bound to the phospholipid moiety in the inner leaflet of the OM, and together with the CTD of the VirB9 proteins, shape the O-layer of the complex^22,23^. The I-layer consists of VirB9 NTDs and also an extended portion of the VirB10 NTDs extending towards the IM^20,21^. The high-resolution structure of a complete OMCC from a minimised T4SS derived from the phytopathogenic *Xanthomonas citri*, has been recently attained^21,24^. The structure, solved by single-particle cryo-electron microscopy (cryo-EM), unveiled the atomic configuration that full-length VirB7, VirB9 and VirB10 proteins adopt to shape the entire OMCC. Interestingly, and despite the close similarities with other minimised T4SS (e.g., *A. tumefaciens* VirB, pKM101 and R388), the VirB7 lipoprotein features a globular N0 domain at its CTD with a fold similar to proteins associated with other bacterial secretion systems^25^. This domain extends out from the central core of the OMCC, thus shaping the “flying saucer” configuration of the complex.

**Fig. 1:**
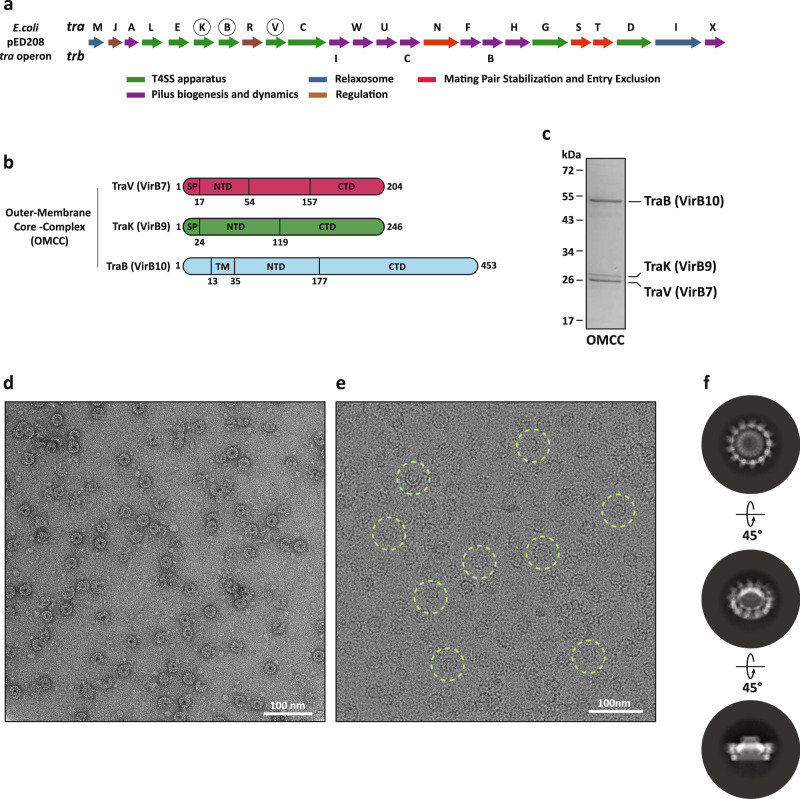
The genetic organisation, biochemistry and EM analysis of the F plasmid outer membrane complex. **a** The native arrangement of T4SS-associated genes within the pED208 *tra* operon with the arrows representing the genes encoding for the T4SS apparatus (green), relaxosome components (blue), mating pair stabilisation and entry exclusion (red), pilus biogenesis and dynamics (purple), and regulation (brown). **b** The primary sequence structure of the three proteins TraV (purple), TraK (green) and TraB (cyan) including their domains and boundaries. **c** SDS-PAGE of the OMCC with the bands of the proteins TraB (VirB10), TraK (VirB9) and TraV (VirB7). **d** Negative stained EM micrograph of the OMCC. Scale bar 100 nm. **e** Cryo-EM micrograph of the OMCC, with particles highlighted with green circles. Scale bar 100 nm. **f** Representative top-, tilted- and side-view 2D class averages obtained using CryoSparc v3.1.0 (see 'Methods' section for detailed information).

The extended conjugative T4SS encoded by members of the F-plasmids (e.g., pED208) are responsible for the emergence of multi-antibiotic resistant in a wide range of pathogenic strains^26^. F plasmid-encoded T4SSs are remarkably more complex than the minimised T4SS counterparts (e.g., virB, pKM101 and R388) and are the only able to extend and retract their pilus during conjugation^2^. The *tra* gene operon within the pED208 plasmid encodes ~25 different proteins (Fig. 1a) involved in different steps of ssDNA transfer during conjugation e.g., T4SS assembly, pilus biogenesis and dynamics, mating pair stabilisation and entry exclusion, relaxosome assembly and regulation^27^. Within the *tra* region, at least ten proteins share homology to the VirB/D system^28^. This includes the three proteins, TraV, TraK and TraB, which assemble the OMCC (F-OMCC), and show homology to VirB7, VirB9 and VirB10, respectively^29^ (Supplementary Fig. 2). Our understanding of how the F-pili assembles at the cell surface and the organisation of the T4SS at the base has been shaped by recent structural advancements which include the atomic structure of the F-pilus and the in situ cryo-electron tomography analysis of the F-T4SS^30,31^. This analysis showed the F-OMCC adopting a 13-fold symmetry; a symmetry that has only been observed in the OMCC structure from the infectious Dot/Icm T4SS from *Legionella pneumophila*^32–34^. Despite these recent advances, we still lack atomic details on the architecture of conjugative OMCCs from both minimal and extended T4SSs. Importantly, these structures will help us decipher mechanistic details of pilus dynamics and DNA transport during conjugation that can be exploited to suppress antibiotic resistance dissemination.

Here, we present the structure of an outer-membrane core complex from the iconic F-T4SS encoded by the *E. coli* pED208 plasmid solved by single-particle cryo-electron microscopy at 3.3 Å resolution. This 2.1 MDa structure, provides an atomic model of the TraB, TraK and TraV proteins and unveils unique stoichiometry relationships between the three proteins. Surprisingly, the complex is formed by two highly flexible concentric rings made by a total of 69 proteins chains arranged in two distinct symmetries. Strikingly, TraK and TraV proteins adopt multiple conformations within one of the complex rings and TraB and TraV can exist in both areas of symmetry. Our model reveals an exceptional protein organisation within the F-OMCC that could account for the high flexibility of the complex. Such flexibility might be pivotal to accommodate the unique dynamic properties (extension and retraction) of the F-pilus during conjugation.

## Results

### Purification and cryo-EM reconstruction of the F-T4SS outer membrane core complex (OMCC)

The outer-membrane core complex (OMCC) proteins TraB, TraK and TraV are part of the F-T4SS encoded in the *tra* region of the conjugative pED208 plasmid from *E. coli*^29^ (Fig. 1a). The three OMCC genes were cloned, over-expressed and the complex purified from *E. coli* membranes using affinity and gel filtration chromatography (Fig. 1b, c). The correct protein bands in the SDS-PAGE gel were verified by LC-MS/MS. Upon purification, the integrity and homogeneity of the complex were accessed by negative-stain EM (Fig. 1d) before vitrification in a thin carbon layer and imaging by cryo-EM (Fig. 1e). A cryo-EM dataset of ~1.45 million particles yielded well-resolved 2D class averages showing different orientations of the complex in the ice (Fig. 1f). Surprisingly, on close inspection of the top/bottom 2D class averages, we could readily identify two distinct areas of symmetries (C17 and C13), one defining a central inner ring (OMCC_IR_) and the other a peripheral outer ring (OMCC_OR_) in the complex (Fig. 1f).

**Fig. 2:**
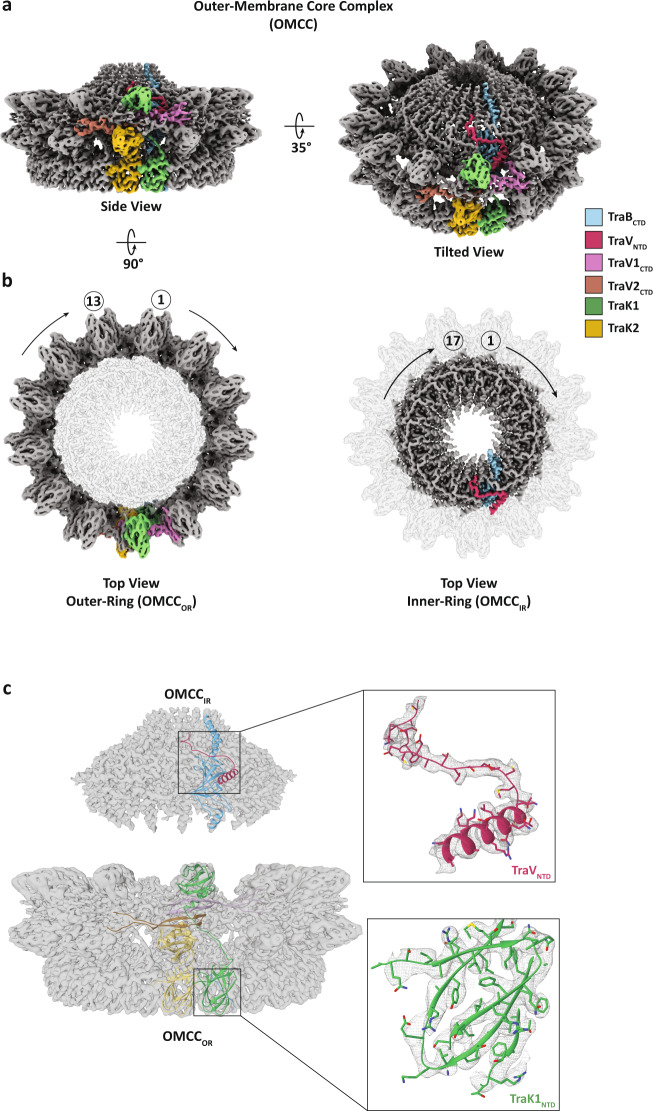
Cryo-EM map and model of the OMCC. **a** Overview of the side- and tilted-view of the electron density map of the complete OMCC with the asymmetric unit highlighted with the colour of the respective individual proteins. **b** Top view of the electron density map of the full OMCC highlighting the outer-ring (left) and inner-ring (right) symmetries and their respective asymmetric unit protein densities. **c** Electron density of the OMCC_IR_ (top) and OMCC_OR_ (bottom) with the asymmetric unit docked into their respective density. The top right shows the refined TraV_NTD_ (purple) model in its density and the bottom right shows the refined TraK1_NTD_ (green) model in its density.

Based on the symmetry information obtained from the 2D class averages, we masked the OMCC_IR_ and OMCC_OR_ regions from our initial low-resolution map, generating two individual high-resolution maps correlating with the C17 (OMCC_IR_) and C13 (OMCC_OR_) rings (Fig. 2a, b). Independent homogeneous refinement of the data yielded two maps with an overall average resolution of 3.3 Å (OMCC_IR_) and 3.4 Å (OMCC_OR_) both extending locally to 3.1 Å (Fig. 2 and Supplementary Fig. 1). Segments corresponding to the OMCC_IR_ asymmetric unit (ASU_IR_) and OMCC_OR_ asymmetric unit (ASU_OR_) were identified in the maps and used to build the atomic model of the entire unit. In the ASU_IR_, an atomic model of the TraB C-terminal domain (CTD) and TraV N-terminal domain (NTD) could be built and refined into the electron density (Fig. 2c). Unexpectedly, the ASU_OR_ segment of the map revealed densities to build and refine more than one molecule of TraK and TraV (Fig. 2a, b). Indeed, densities to fit two NTD and CTD domains of TraK (TraK1 and TraK2) were identified. Similarly, densities corresponding to two TraV_CTD_ (TraV1_CTD_ and TraV2_CTD_) were also observed in the ASU_OR_ segment. An additional streak of density adjacent to the TraK1_NTD_ density was noticed in the inner wall of the OMCC_OR_ channel corresponding to TraB residues 177–186 (Supplementary Fig. 3b).

### The atomic structures of the OMCC_IR_ and OMCC_OR_ asymmetric units

**Fig. 3:**
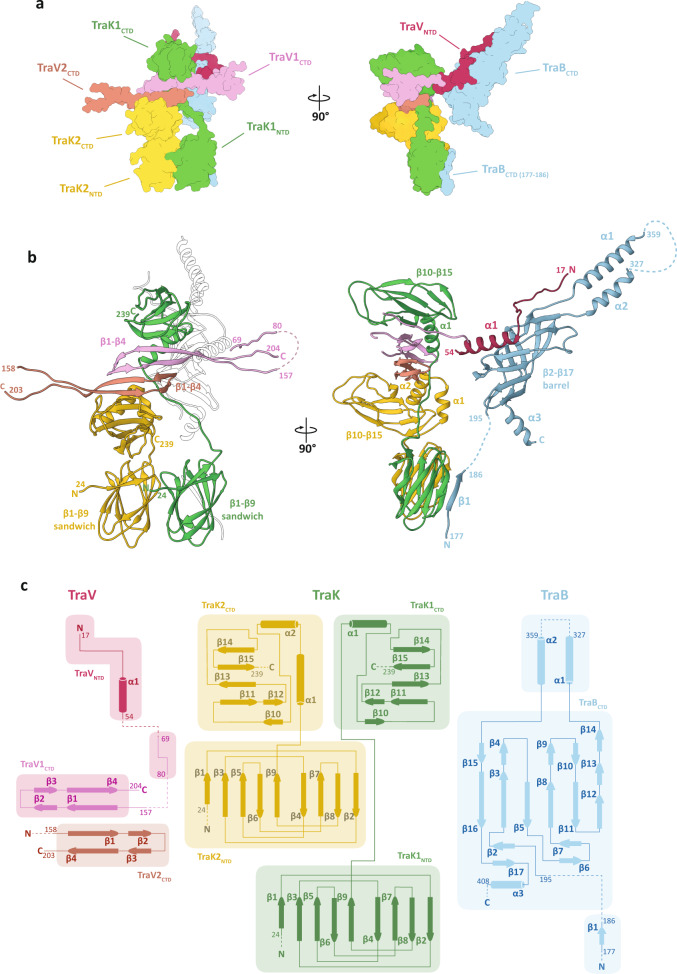
The combined asymmetric units of the OMCC. **a** The side- and a 90° rotated view of the combined OMCC asymmetric units (ASU_OR_ and ASU_IR_) in surface representation, highlighting individual protein and protein domains. Individual proteins colour coded as described in Fig. 2a. **b** Cartoon representation of the combined ASU (ASU_OR_ and ASU_IR_) with same views as in ‘**a**’, colour coded as in Fig. 2a. The individual domains and key secondary structural features along with the protein boundaries are highlighted. **c** Topology secondary structure diagrams of TraV (purple, pink and brown), TraK (yellow and green) and TraB (cyan). β-strands and α-helices are represented as arrows and cylinders, respectively. Regions for which no electron density was observed are indicated by dashed lines.

Models of the TraB_CTD_ and TraV_NTD_ protein chains were built in the OMCC_IR_ map, while models for TraK1, TraK2, TraV1_CTD_, TraV2_CTD_ and TraB_CTD(177-186)_ were built in the OMCC_OR_ map (Fig. 3a). A significant proportion of the ASU_OR_ is constituted by TraK where two chains are present per ASU. The TraK_NTD_ is made up of nine β strands (β1 – β9) in two sheets making a β sandwich domain arrangement (Fig. 3b, c). Two such domains from the respective TraK chains (TraK1 and TraK2) are present in a front-to-back arrangement and extensive salt bridge, hydrogen bond and hydrophobic interactions stabilise their interaction (Supplementary Table 1). The structure of the TraK NTD shows the highest degree of structural homology with its pKM101 TraO_CTD_ and *X. citri* VirB10_NTD_ counterparts (Supplementary Fig. 5). The TraK_CTD_ is made of two α helices (α1 and α2) and six β strands (β10 – β15) (Fig. 3b, c). For TraK2, the NTD and CTD are connected by a short linker, essentially placing the CTD closer to the NTD. In the case of TraK1 however, the NTD and CTD are further apart connected by an extended linker that is achieved by the melting of α1 in TraK2 (Fig. 3b, c). The two TraK CTDs are arranged with TraK1 on top of TraK2 and are related by a P2 symmetry. These two TraK CTDs are held together by two anti-parallel TraV chains that wraps the stem of the TraK linkers (α1 – TraK2 and α2 – TraK1) (Supplementary Fig. 3a). TraV has four β strands per chain making two continuous β-sheets with TraV1 and TraV2 that stabilise the entire OMCC_OR_ complex (Fig. 3b, c and Supplementary Fig. 3a). A short linker sequence (residues 69–80) assigned to TraV1 and positioned adjacent to TraK1_CTD_, provides one of the key connections between the inner and the outer rings of the OMCC (Fig. 3b, c). The N-terminal end of the TraV (residues 17–54) is located entirely in the inner ring which includes a single helix α1 (Fig. 3b, c and Supplementary Fig. 3c). The structure of TraV appears to be unique and does not show structural homology with any of the reported OMCC protein structures. The ASU_IR_ is predominantly made up of TraB_CTD_ which includes a total of seventeen β strands and three α helices. Six of these strands (β3, β5, β8, β14–β16) mediate the interaction with TraV_NTD_ (Supplementary Fig. 3c). The core of the structure has a β barrel that connects to two anti-parallel α helices (α1 and α2) and a third helix (α3) towards the C-terminal end. The structure of TraB_CTD_ is highly conserved amongst the closest homologues of TraB (Supplementary Fig. 5), as are the protein sequences within this region (Supplementary Fig. 2a), thus making it an essential structural feature in the T4SS architecture^21,28^. TraB also extends into the outer ring with a short stretch of sequence (residues 177–186) at the N-terminal end of the CTD. This short sequence folds into a β-strand eventually adding to one of the sheets within the TraK1 β sandwich (Fig. 3b, c and Supplementary Fig. 3b). Details of the residues that mediate the interaction between each of the protein chains within the ASU_IR_ and ASU_OR_ can be found in Supplementary Table 1.

### Interactions network between neighbouring ASUs in the OMCC_IR_ and OMCC_OR_

**Fig. 4:**
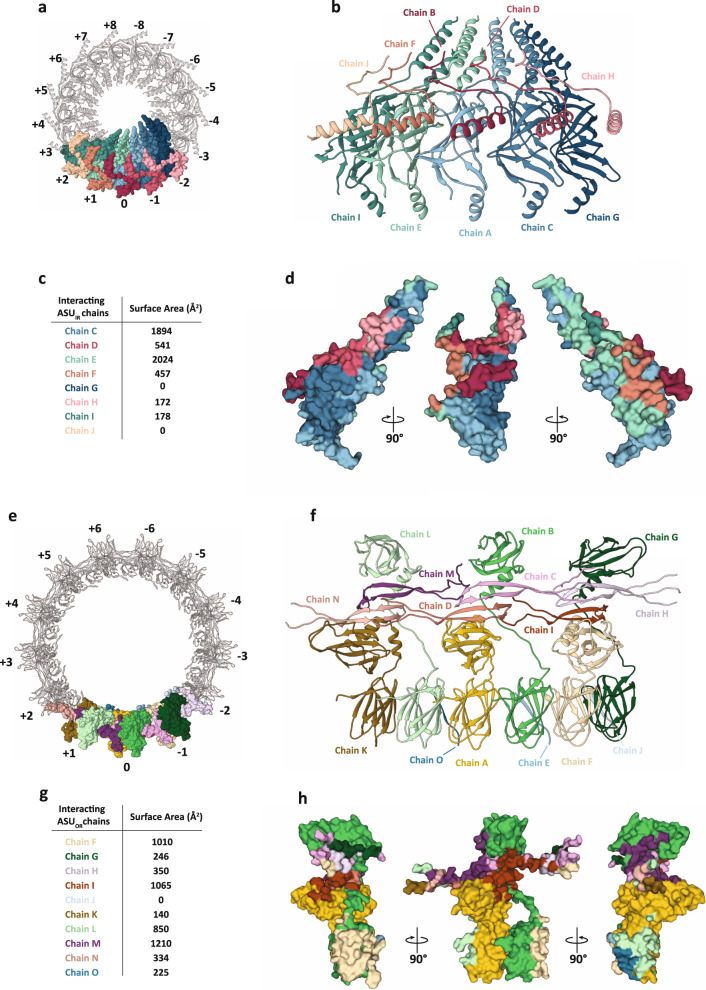
Interaction between ASUs in the OMCC. **a** Top view of the OMCC_IR_ with the surface representation of five ASUs, the central ASU-0_IR_ and its interacting ASUs +1, +2, −1 and −2. The TraB and TraV of ASU-0_IR_ are colour coded as described in Fig. 2a. TraB of the +1, +2, −1 and −2 ASUs are coloured in light green, dark green, light blue and dark blue, respectively, and TraV of the +1, +2, −1 and −2 ASUs are coloured in brown light brown, pink and light pink, respectively. **b** Side view of the five ASU in cartoon representation colour coded as described in ‘**a**’. **c** Surface area buried in ASU-0_IR_ by interacting protein chains. **d** Mapping of interacting chain-ASU interactions onto ASU-0_IR_. The interactions made by the neighbouring ASU chains are mapped onto ASU-0_IR_ using the same colour code as ‘**a**’. **e** Top view of the OMCC_OR_ with the surface representation of three ASUs, the central ASU-0_OR_ and its interacting ASUs +1 and −1. The TraB, TraK and TraV of ASU-0_OR_ are colour coded as described in Fig. 2a. TraB of the +1 and −1 ASUs are coloured as dark blue and light blue respectively, TraK1 of the +1 and −1 are coloured in light green and dark green, TraK2 of the +1 and −1 are coloured in dark brown and light yellow, respectively, TraV1 of the +1 and −1 are coloured in dark pink and light pink, TraV2 of the +1 and −1 are coloured in light orange and brown, respectively. **f** Side view of the three ASU in cartoon representation colour coded as described in ‘**e**’. **g** Surface area buried in ASU-0_OR_ by interacting protein chains. **h** Mapping of interacting chain-ASU interactions onto ASU-0_OR_. The interactions made by the neighbouring ASU chains are mapped onto ASU-0_OR_ using the same colour code as ‘**e**’.

The ASUs both at the inner and outer ring make extensive contact with its adjacent units stabilising the entire OMCC structure. In the inner ring, seventeen ASU_IR_ heterodimers made of TraB_CTD_ and TraV_NTD_ (labelled −8 to +8 in the clockwise direction) assemble to form the complete OMCC_IR_ (Fig. 4a). A single ASU_IR_ makes contact with four other neighbouring ASUs, involving two ASUs each in the clockwise and anti-clockwise direction (Fig. 4a, b). The central reference ASU_IR_ numbered 0, (chains A and B) interacts with ASUs +1 (chain E and F), ASU + 2 (chain I and J), ASU-1 (chain C and D) and ASU-2 (chain G and H) (Fig. 4b). ASU + 2 bury a surface area of 178 Å^2^ whereas ASU-2 bury a surface area of 172 Å^2^ on ASU-0_IR_. The ASUs +1 and −1 are contiguous to ASU-0_IR_ and hence bury a much larger surface area of 2481 Å^2^ and 2435 Å^2^, respectively (Fig. 4c, d). Extensive inter ASU interactions stabilise the OMCC_IR_ including salt bridge, hydrogen bond and hydrophobic interactions (Supplementary Table 2). Notably, an inter TraV_NTD_ di-sulphide bond has been observed between residue Cys35 of chain B and Cys27 of chain D. This interchain di-sulphide bond between contiguous TraV_NTD_ chains creates a wide mesh that spreads over the TraB proteins and extends to the periphery of the OMCC_IR_ (Supplementary Fig. 4). The cysteine residues involved in the di-sulphide bond are only conserved in TraV homologues belonging to the F-T4SS family (Supplementary Fig. 2c, d). In addition, the lipidated and highly conserved Cys18 of chain B is stabilised by interaction with Tyr366 of chain I through its main chain oxygen l^35^ (Supplementary Figs. 2c, d and  4). For the outer ring, thirteen ASU_OR_ heteropentamers made of TraK1, TraK2, TraV1_CTD_, TraV2_CTD_ and TraB_CTD(177-186)_ (labelled −6 to +6 in the clockwise direction) assemble to form the complete OMCC_OR_ (Fig. 4e). Each ASU makes contact with another ASU both in the clockwise and anti-clockwise direction. The central reference ASU_OR_ numbered 0, (chains A, B, C, D and E) interacts with ASU + 1 (chains K, L, M, N and O) and ASU-1 (chains F, G, H, I and J) (Fig. 4f). ASU_ORs_ + 1 and −1 are contiguous to ASU-0_OR_ and bury a surface area of 2759 Å^2^ and 2671 Å^2^, respectively (Fig. 4g, h). Extensive interactions between contiguous TraK_NTDs_ from adjacent ASUs stabilise the OMCC_OR_. These inter ASU TraK_NTD_ interactions are identical to the intra ASU interactions observed between TraK1 and TraK2 NTDs (chains A and E) (Fig. 4f and Supplementary Tables 1 and 2). Adjacent ASUs are also stabilised by interactions between TraV CTDs which include mostly hydrophobic interactions between chain C and H, and between chain D and I. In addition, hydrogen bond interactions are also observed between chain C and H and between chain D and I (Fig. 4f).

### The architecture of the complete F-OMCC

**Fig. 5:**
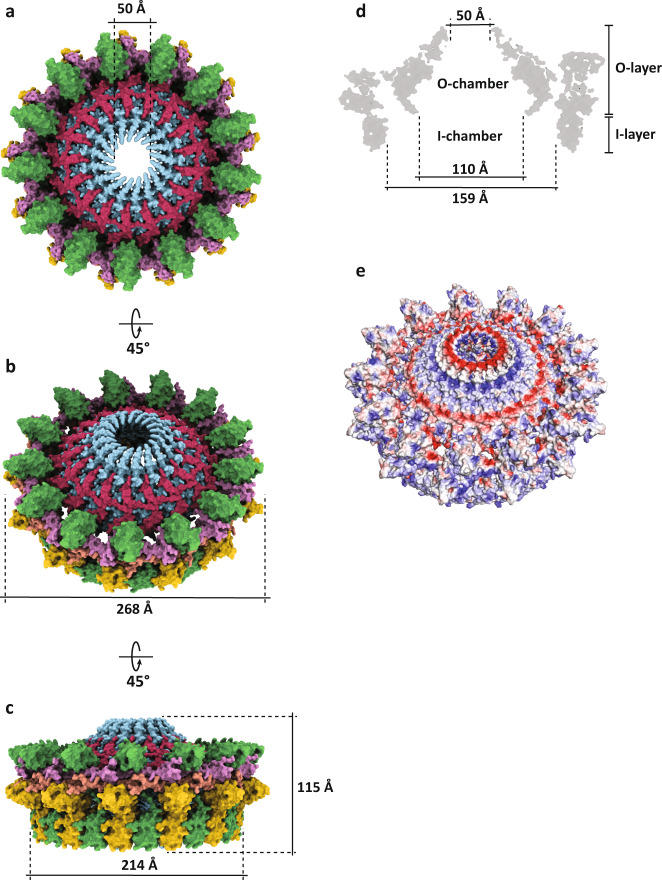
Structure of the outer membrane core complex. **a** Top view of the structure of OMCC in surface representation with each protein within the complex coloured in their respective colours. **b** Tilt view of the structure in surface representation with the width of the O-layer. **c** Side view of the structure in surface representation with the overall height of the complex and the width of the I-layer. **d** Cut-away side view of the model with the dimensions at distinct positions of the inner channel and indicating specific structural features within the complex. **e** Distribution of surface charges on the OMCC structure.

The interactions described in the above sections contribute towards the oligomeric assembly of the two rings to form the complete F-OMCC. The inner ring is made of seventeen chains of TraB and TraV proteins each, making a total of 34 chains. The outer ring is made of 26 chains of TraK and TraV each, and 13 chains of TraB, making a total of 65 chains. The inner and outer ring are intricately connected, where 13 of the 17 TraB chains belonging to the inner ring contact the inside surface of the outer ring. The seventeen of the 26 TraV chains of the outer ring extend into the inner ring stabilising the OMCC through a wide protein mesh described earlier (Supplementary Fig. 4). This unusual arrangement between the two OMCC rings ultimately make the complete F-OMCC constructed by 69 polypeptide chains. This complex has overall dimensions of 115 Å in height, 268 Å in diameter and a narrow 50 Å exit channel shaped by TraB α1 and α2 helices (Fig. 5a–c). The TraB α1 helix and TraV NTD residues Gly17-Ser23 define a rim-ward hydrophobic surface suggesting that this portion of the OMCC is likely embedded in the bacterial OM (Fig. 5e). The hydrophobic rim-ward defined mostly by TraB α1 has a height of ~30 Å and is in agreement with the hydrophobic surface height observed in the O-layer of the functionally related pKM101 T4SS^19^. Conjugative OMCCs seem to be part of a small number of alpha-helical embedded outer-membrane complexes in bacterial secretion systems^5^. The proteins TraB_CTD_, TraV, TraK_CTD_ define the O-layer and the TraK_NTDs_ define the I-layer regions of the complex (Fig. 5d). The complex makes a central hollow segment with two chambers, a lower I-chamber outlined by the I-layer and a conical O-chamber outlined by the O-layer (Fig. 5d). The conical O-chamber has an internal diameter of 110 Å at the periplasmic side and constricts into an exit channel towards the outer membrane (Fig. 5d). The diameter of the exit channel (50 Å) is too narrow to accommodate the 87 Å external diameter of the F-pilus, thus our structure likely represents the F-OMCC in its closed state. It is noteworthy that the exit channel is likely to undergo structural rearrangements to accommodate a growing pilus during pilus biogenesis.

### TraV and TraB facilitate flexible contacts between the OMCC_IR_ and the OMCC_OR_

**Fig. 6:**
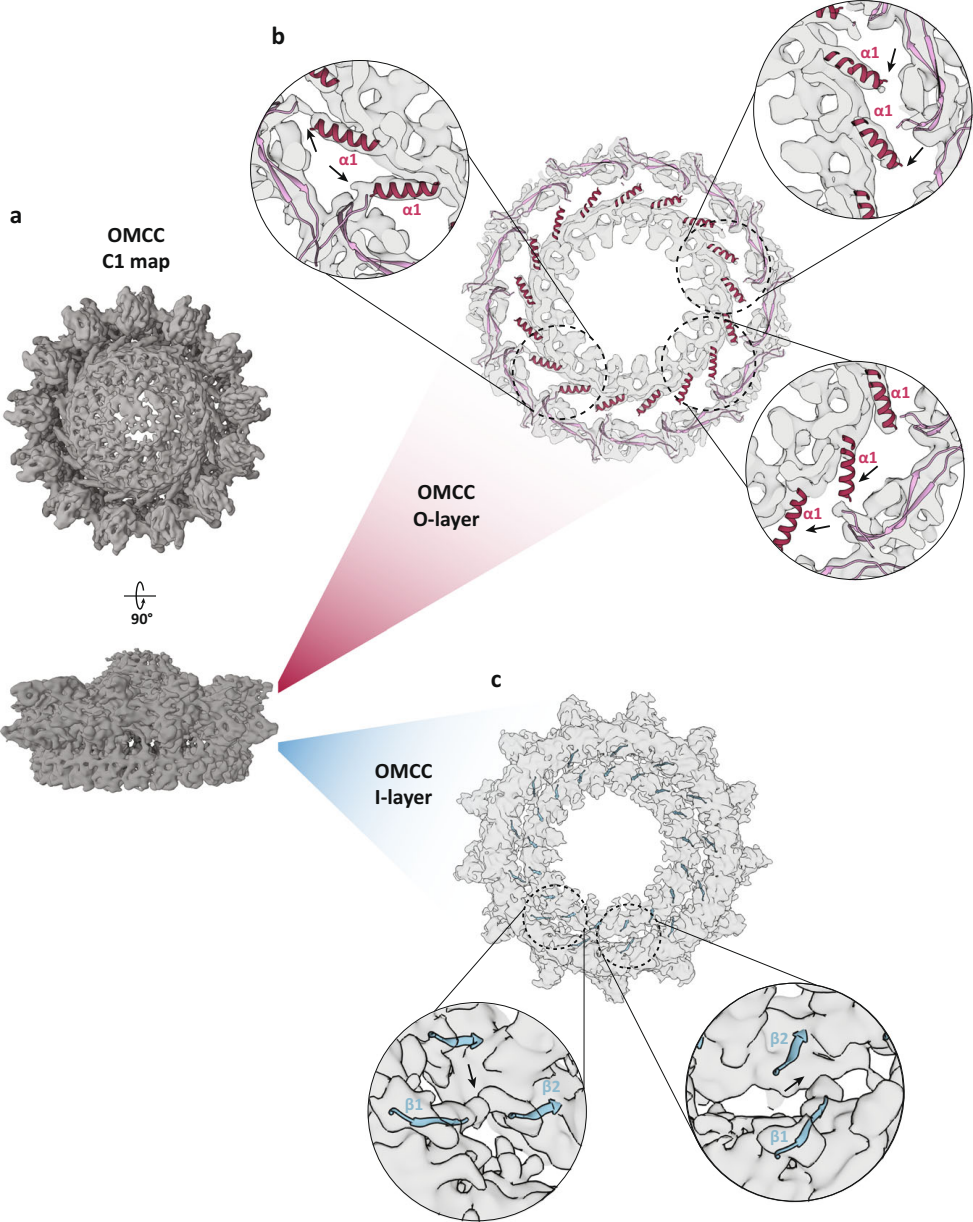
OMCC asymmetric map and complex flexibility. **a** Overview of the top and side views of the OMCC electron density map reconstructed without any imposed symmetry determined at a resolution of 5.7 Å. **b** Horizontal slice of the OMCC C1 map (contour level 0.34) taken at the indicated O-layer location. Each inset shows different levels of electron densities (indicated by arrows) in the connections between the TraV1_CTD_ linker (residues 69–80) located at the OMCC_OR_ and the TraV α1 helix located at the OMCC_IR_. **c** Horizontal slice of the OMCC C1 map (contour level 0.16) taken at the indicated I-layer location. Each inset shows different levels of electron densities (indicated by arrows) in the connections between the TraB β1 located at the OMCC_OR_ and TraB β2 located at the OMCC_OR_.

In order to address the symmetry mismatch between the OMCC_IR_ and the OMCC_OR_ we processed the same dataset of particles and generated a 3D map without applying any symmetry parameters (Table 1). This unsymmetrized map showed clear C13 and C17 symmetries matching the position of the outer and inner ring models obtained from the symmetrized maps (Fig. 6a). This map has an average resolution of 5.7 Å to which we were able to dock the atomic model of the OMCC_IR_ and the OMCC_OR_. Although most parts of the two models fit into the map density, some radial segments at the interface between the OMCC_IR_ and the OMCC_OR_ lack densities (Fig. 6b, c). At the O-layer, connections between the inner and outer ring of the OMCC are established by a flexible stretch of 14 amino acids (residues 55–68) between the TraV α1 and TraV1_CTD_ linker (residues 69– 80) that are invisible in the C1 map. This lack of density implies a high degree of flexibility between both rings mediated by this O-layer flexible linker. This flexibility also extends onto the α1 helix where some protomers exhibit variable degrees of electron density in the C1 map suggesting possible rotational movement between the two OMCC rings (Fig. 6b). The OMCC inner- and outer rings are also connected by a proline-rich eight residue linker (residues 187–194) connecting TraB β1 and β2 at the I-layer. A continuous density corresponding to this linker can be observed for a few protomers in the C1 map, however, most of the linker connections are absent in the same map (Fig. 6c). This absence of density for the I-layer linkers again suggests some degree of flexibility between the two OMCC rings exists. Overall, flexibility controlled at two independent locations (I-layer and O-layer), suggest a highly dynamic complex capable of adapting to pilus extension and retraction during conjugation.

**Table 1:** Cryo-EM data collection, processing and model refinement statistics.

	OMCC inner ring	OMCC outer ring	OMCC C1
Data collection and processing			
Microscope	Titan Krios	Titan Krios	Titan Krios
Camera	K3	K3	K3
Voltage (kV)	300	300	300
Electron exposure (e^-^ Å^−2^)	50	50	50
Pixel size (Å)	1.08	1.08	1.08
Defocus range (μm)	−0.7 to −3.0	−0.7 to −3.0	−0.7 to −3.0
Symmetry imposed	C17	C13	C1
Initial particle images	1,445,958	1,445,958	1,445,958
Final particle images	74,956	298,235	95,177
FSC threshold	0.143	0.143	0.143
Map resolution (Å)	3.3	3.4	5.7
Map B factor	−74	−93	−304
EMDB code	EMD-12962	EMD-12963	EMD-13231
Refinement and model validation			
Model versus data CC (mask)	0.72	0.75	n/a
Clash score	13.73	13.82	
MolProbity score	2.18	2.25	
Bond lengths rmsd (Å)	0.007	0.005	
Bond angle rmsd (°)	1.136	1.033	
Poor rotamers (%)	0	0	
Ramachandran			
Favoured (%)	90.7	88.03	
Allowed (%)	9.3	11.97	
Outlier (%)	0	0	
PDB code	7OKN	7OKO

## Discussion

The structure of the OMCC from the iconic F plasmid reported here reveals the atomic model of a complete outer membrane core complex from a conjugative secretion system. The components that make up this complex include the proteins TraB, TraK and TraV which are homologous to VirB10, VirB9 and VirB7 respectively and are observed throughout the multi-functional T4SS spectrum^28,29^. Among the conjugative T4SS structures reported so far, all OMCCs have been shown to adopt an homogeneous C14 symmetry throughout the complex^18–20^. However, protein translocating T4SSs have been shown to assemble OMCCs with either homogeneous C14 symmetry as the bacterial killing system (i.e., *X. citri*)^21^, or variable symmetries of C13:C18 (*L. pneumophila*)^33,34^ and C14:C17 (*H. pylori*)^36,37^ between different complex layers. In our complex, however, a mismatch in symmetry is observed between two concentric OMCC_IR_ (C17) and OMCC_OR_ (C13) rings located within the same layer of the complex. This unique arrangement was concealed in the previous OMCC_pED208_ cryo-ET map due to low resolution and the imposed C13 symmetry during reconstruction. This arrangement may contribute to remarkable differences in protein stoichiometry within the complex when compared to other OMCCs^17–21,33,34,36,37^. Specifically, TraB, TraK and TraV are stoichiometrically related as 1:1.53:1.53 with a total of 17:26:26 protein copies to shape the 2.1 MDa F-OMCC structure. This unusual stoichiometry could be rationalised by the differences in the symmetry between the OMCC_IR_ and OMCC_OR_, where 1:1 (TraB:TraV) per ASU exists in the OMCC C17 inner ring and a 1:2:2 (TraB:TraK:TraV) per ASU exists in the OMCC C13 outer ring. It is particularly noteworthy that the OMCC_OR_ C13 symmetry could be expanded to C26 due to the presence of 26 copies of TraK_NTDs_ which make up the complete I-layer ring.

Within the structure, TraK adopts two distinct conformations where the NTDs and CTDs are connected by visible linkers physically connecting the I- and O-layer. In other OMCC structures, the absence of equivalent linker density from TraK homologues has been attributed to the axial flexibility between the I- and O-layer^21^. However, the F-OMCC shows to be rigid between the I- and O-layer with its flexibility provided by the radial TraV connections between the inner and the outer rings. The position of these TraV flexible linkers suggest that non-synchronised rotational movements could happen as a consequence of the dynamic freedom between the two rings. In addition, TraV has a central role in scaffolding the F-OMCC structure by creating a large mesh at the inner ring and a continuous belt holding all the TraK CTDs at the outer ring. Central in maintaining the integrity of this large mesh and thus OMCC symmetry, is the interchain di-sulphide bond interactions established by TraV_Cys27_ and TraV_Cys35_. These TraV cysteine residues, only conserved in members of the F-T4SS family (Supplementary Fig. 2d), are responsible for the correct localisation of TraV to the outer membrane, efficient DNA conjugation to the recipient cell and sensitivity to bacteriophage infection^35^. TraV is also key in providing an interaction hub to other Tra/Trb proteins (e.g., TraH, TraF, TraW and TrbC) to the OMCC^38,39^. The presence of these interacting partners could stabilise the flexible regions of TraV for which we did not assign a sequence. TraB is the most structurally conserved protein at the O-layer, and interactions at the I-layer have only been observed in OMCCs structures of *X. citri* and F-OMCC^21^. In the case of the *X. citri* OMCC a protein helix was observed at the base of the I-layer, but the linkers connecting to the rest of the VirB10 proteins at the O-layer were absent. However, in the F-OMCC a continuous density could be observed for a few TraB protein chains suggesting that the eight residues proline-rich sequence between the β1- and β2-strands could indeed connect both strands (Supplementary Fig. 2a). Yet, this density could not be observed for most of the TraB proteins implying that the proteins experience variable levels of flexibility in the outer ring channel surface. This lack of density could be either explained by translational movements of the protein chains along the OMCC_OR_ channel surface or, by a “sliding” mechanism that changes the protein register to accommodate rotational movements undertaken by the OMCC_IR._ Notably, the interaction between TraB β1 and TraK β4 strands are mediated exclusively by main chain interactions possibly suggesting transient contacts between the two proteins (Fig. 3 and Supplementary Fig. 3b). The location of TraB β1 in the inner side of the OMCC_OR_ was not entirely unexpected and is in agreement with previous observations in the *X. citri* OMCC structure, where a section of VirB10 (residues 150–161) contacts the inner wall of the OMCC I-layer^21^. The interaction between TraK and TraB was reported previously^29^. Yeast-two-hybrid results show that a TraB region incorporating the β1 residues, is the only region capable of interacting with the β4 of TraK^29^. Overall, the extensive flexibility observed at various regions of the OMCC, dictated by the TraB and TraV proteins, suggest a highly dynamic complex capable of adapting to an emerging F-pilus during biogenesis and, also playing a central role during filament extension and retraction.

In summary, we report the high-resolution structure of a conjugative T4SS outer membrane core complex encoded by the iconic F plasmid. The structure markedly differs from previously reported OMCCs due to its unique structural organisation with two concentric rings related by mismatched symmetries, radial structural flexibility and defined connections between the O- and I-layer of the complex. Overall, the structure provides exciting new insights into the dynamics mediating F-pilus extension/retraction and ultimately DNA transfer during conjugation. This work sets the stage for future investigations aiming to understand the mechanistic details of antimicrobial resistance gene transfer among bacteria.

## Methods

### Outer membrane core-complex expression and purification

The sequence coding for *traB-strep, traK* and *traV* was cloned as a polycistronic operon into pBAD24 vector and transformed into TOP10 cells. Protein overexpression was induced at OD_600_ = 0.6 by addition of 0.2% w/v arabinose for 18 h at 18 °C. The cells were harvested by centrifugation at 7000 × *g*, 20 min at 4 °C, and the pellet was resuspended in 50 mM Tris-HCl (pH 7.5), 200 mM NaCl, 1 mM EDTA, 0.2 mg ml^−1^ lysozyme, protease inhibitor tablet. Following lysis by sonication on ice, cellular debris was removed by centrifugation at 35,000 × *g* for 20 min. The crude membrane fraction was isolated by ultracentrifugation at 120,000 × *g* for 1 h. Membrane proteins were solubilised with gentle agitation for 1 h in 50 mM Tris-HCl (pH 7.5), 300 mM NaCl, 1 mM EDTA, 0.8% w/v LDAO (Anatrace) and 1% w/v DDM (Anatrace). The insoluble material was pelleted by ultracentrifugation at 100,000 × *g* for 40 min and the supernatant was loaded onto a Strep-Tactin Sepharose affinity column (GE Healthcare). The column was washed with 50 mM Tris-HCl (pH 7.5), 300 mM NaCl and 0.23% w/v LDAO and, the protein eluted in the same buffer supplemented with 10 mM d-desthiobiotin (IBA). The eluted proteins were loaded onto a Superose 6 GL 10/300 (GE Healthcare) equilibrated with 50 mM Tris-HCl (pH 7.5), 300 mM NaCl and 0.23% w/v LDAO, following concentration by centrifugal concentrator with 100 kDa molecular weight cut-off. The proteins were analysed by SDS-PAGE and Coomassie blue staining.

### Electron microscopy sample preparation and data collection

To assess the F-OMCC purity and homogeneity after size-exclusion chromatography (SEC), 10 µl of protein sample was applied onto glow-discharged carbon-coated copper grids (300 mesh, Agar Scientific). After incubation for 2 min at room temperature, grids were washed twice with 10 µl of water, blotted with filter paper and stained for 1 min with 2% w/v uranyl acetate. Excess stain was removed by a final blotting step and grids were let to dry before imaging. Negative-stain (NS) images of the F-OMCC were acquired on a FEI Tecnai G2 Spirit operating at 120 kV equipped with a LaB_6_ filament and a CCD 4k × 4k camera. For F-OMCC structure determination, 4 µl of the sample, previously assessed by NS, was adjusted to 0.1 mg/ml and applied onto glow-discharged Lacey copper grids with ultrathin carbon film (300 mesh, Agar Scientific). Samples were vitrified in liquid ethane using a Leica automatic plunge freezer EM GP2 with a 2.4–2.8 s blot time under 85% relative humidity at 4 °C. The frozen girds were checked for sample concentration and ice thickness using the Jeol 2100 plus microscope. The grids with ideal protein concentration and ice thickness were used for data acquisition at LonCEM (London Consortium EM, Francis Crick Institute) using a FEI Titan Krios operating at 300 kV. Images were collected automatically using EPU software (FEI) on a Gatan K3 detector in counting mode with a pixel size of 1.08 Å. A total of 11588 movies were recorded with a nominal defocus range of ~−0.7 to −3.0 µm. Each image consisted on a movie stack of 38 frames with a total dose of 50 e^-^/Å^2^ over 3.9 s corresponding to a dose rate of 15 e^-^/px/s.

### Cryo-EM image processing and reconstruction

All image processing was done with CryoSparc^40^ (v3.1.0) unless otherwise mentioned. The movie stacks were aligned and summed using patch motion correction, and CTF was estimated using the patch CTF estimation function. After screening the micrographs for good Thon Rings and ideal ice thickness, 10,911 images remained in the dataset for further processing. In total, 3506 particles were manually picked and classified to generate 2D class references for automated particle picking of the entire dataset. After establishing the correct auto-picking parameters using a small number of images, a total of 1,445,958 particles were auto-picked from the entire dataset and extracted using a box size of 480 × 480 pixels. Low-quality particles were removed after several rounds of 2D classification, resulting in a stack of 298,235 particles with defined OMCC_OR_ features. Further rounds of 2D classification, improved the visual details of the OMCC_IR_ and resulted on a final stack of 74,956 particles. After visual inspection of the 2D classes, we determined that we had two different symmetries in the complex, C13 for the OMCC_OR_ and C17 for the OMCC_IR_. Ab-initio maps were generated and all OMCC_OR_ particles were subjected to 3D homogeneous refinement (with C13 symmetry applied) with a solvent mask (5 pixels extension and 6 pixels of drop off) encompassing the OMCC_OR_. Similarly, all OMCC_IR_ particles were subjected to the same 3D procedure, this time with C17 symmetry applied to the inner-ring with a solvent mask with five pixels extension and six pixels of drop off applied. Simultaneously, 95177 particles were used to generate an unsymmetrized map confirming the existence of the C13 and C17 symmetries at OMCC_OR_ and OMCC_IR_ respectively. Further, a B factor of −74, −93 and −304 was applied to the OMCC_IR_, OMCC_OR_ and OMCC_C1_ maps, improving the overall resolution to 3.3 Å, 3.4 Å and 5.7 Å (FSC = 0.143), respectively. The local resolution estimation in the OMCC_OR_ and OMCC_IR_ maps was performed in Relion 3.1^41^. Statistics for data collection and 3D refinement are included in Supplementary Table 1.

### Model building and refinement

Model building started with identifying the single asymmetric unit (ASU) in both the OMCC_OR_ and the OMCC_IR_ maps and building into the density using both maps. For the inner ring once the ASU was identified, single helices were placed manually into the density using Coot^42^. Using these helices as templates, the remainder of the structure was built manually by extending the chains at both the N and C-terminus. Once a single unit of TraB was built, the various chains were merged to make a single chain of TraB protein. The OMCC_IR_ ASU had an extra density corresponding to an alpha helix. A poly-alanine helix was placed into the density and the chain was extended at the N-terminus till the end of the continuous density. First, bulky side chains were assigned and then, by identifying connecting sequences, the N-terminus of the TraV was built in the density. The correct side chains were assigned to the poly-alanine chain, thereby allowing a single ASU of the OMCC_IR_ to be built. This central ASU was copied and manually placed to occupy two clockwise and anti-clockwise unit densities. The resulting ASU, + 1, +2, −1 and −2 model was used for refining the central ASU. The model was subjected to several iterative rounds of real space refinement using Phenix^43^ and progress in refinement was tracked using Ramachandran plot and Molprobity^44^. After successful refinement of the central ASU model, a PDB file with two adjacent ASUs were generated by fitting into their respective density using CHIMERA^45^. The rotation and translation parameters of these two chains were then calculated using the programme LSQKAB^46^. A PDB coordinate file was then generated using the rotation and translation parameters obtained with the programme PDBSET^47^ with seventeen ASUs making the full inner ring structure.

In the case of the outer ring, the VirB9 model of the *X. citri* OMCC structure^21^ was used as a template to build into the density. A single TraK1_NTD_ was manually built using Coot^42^ and this was copied to fit into the TraK2_NTD_ density part of the same ASU. The TraK2_CTD_ was built first by extending the chain from the I-layer and placing the poly-alanine alpha helix into its respective density. Once the TraK2_CTD_ chain was built, a copy of the same chain was placed into the respective density in the TraK1_CTD_. Clear continuous density was visible between the TraK1_NTD_ and TraK1_CTD_ densities wherein a poly-alanine chain was manually built. Amino acids with bulky side chains were assigned first and by identifying the connecting sequence between two bulky residues the correct sequence was assigned. This was built further to occupy the continuous density thereby, resulting in two TraV chains. Also, a TraV sequence was built into a connecting density adjacent to the TraK1_CTD_. Finally, an extra density radially located inside of the I-layer section of the outer ring was assigned to TraB and built manually using Coot^42^. Once the entire ASU was built, it was copied and manually placed to occupy one ASU clockwise and one ASU anti-clockwise unit densities. This ASU, + 1 and −1 was used to refine the central ASU chains. These three ASU unit was subjected to several rounds of real space refinement using Phenix^43^ and the progress was monitored using Ramachandran plot and Molprobity^44^. Once the ASU was successfully refined, a PDB coordinate file containing two adjacent ASUs was created by fitting the central ASU model into two adjacent densities using Chimera^45^. Similar to the inner ring, the rotation and translation parameters were calculated using LSQKAB^46^ and a coordinate file with thirteen ASUs were generated using PDBSET^47^. Two separate coordinate files corresponding to the OMCC_IR_ and OMCC_OR_ were submitted to the protein data bank with the entry codes 7OKN and 7OKO, respectively.

### Structure analysis and presentation

The analysis and visualisation of the cryo-EM maps and atomic models were done using Chimera^45^, ChimeraX^48^, PyMol (Molecular Graphics System, Schrödinger), and PDBePISA (https://www.ebi.ac.uk/pdbe/pisa/).

### Reporting summary

Further information on research design is available in the Nature Research Reporting Summary linked to this article.

## Supplementary information

Supplementary Information

Reporting Summary

## Data Availability

The data that support this study are available from the corresponding authors upon reasonable request. The atomic coordinates have been deposited at the Protein Data Bank with accession codes 7OKN (OMCC_IR_) and 7OKO (OMCC_OR_). The density maps have been deposited at the Electron Microscopy Data Bank with accession codes EMD-12962 (OMCC_IR_), EMD-12963 (OMCC_OR_) and EMD-13231 (OMCC_C1_).
