# Publisher Correction: Architecture of the outer-membrane core complex from a conjugative type IV secretion system

**DOI:** 10.1038/s41467-022-28181-3

**Published:** 2022-01-31

**Authors:** Himani Amin, Aravindan Ilangovan, Tiago R. D. Costa

**Affiliations:** 1 MRC Centre for Molecular Bacteriology and Infection, Department of Life Sciences, Imperial College, London, SW7 2AZ UK; 2 School of Biological and Chemical Sciences, Queen Mary University of London, London, E1 4NS UK

**Keywords:** Membrane proteins, Bacterial secretion, Cryoelectron microscopy

Correction to: *Nature Communications* 10.1038/s41467-021-27178-8, published online 25 November 2021.

In this article the hyperlinks provided for Protein Data Bank accession codes 7OKN and 7OKO in the Data availability statement were incorrect. The original article has been corrected.

