# Supplementary material for: Architecture of the outer-membrane core complex from a conjugative type IV secretion system
Source: Nat Commun. 2021 Nov 25;12:6834. doi: 10.1038/s41467-021-27178-8 (PMC8617172; doi:10.1038/s41467-021-27178-8)
Supplement: Supplementary file 1 — Supplementary Information [file 41467_2021_27178_MOESM1_ESM.docx]

**SUPPLEMENTARY MATERIAL**

Architecture of the outer-membrane core complex from

a conjugative type IV secretion system

Himani Amin, Aravindan Ilangovan and Tiago R. D. Costa

**
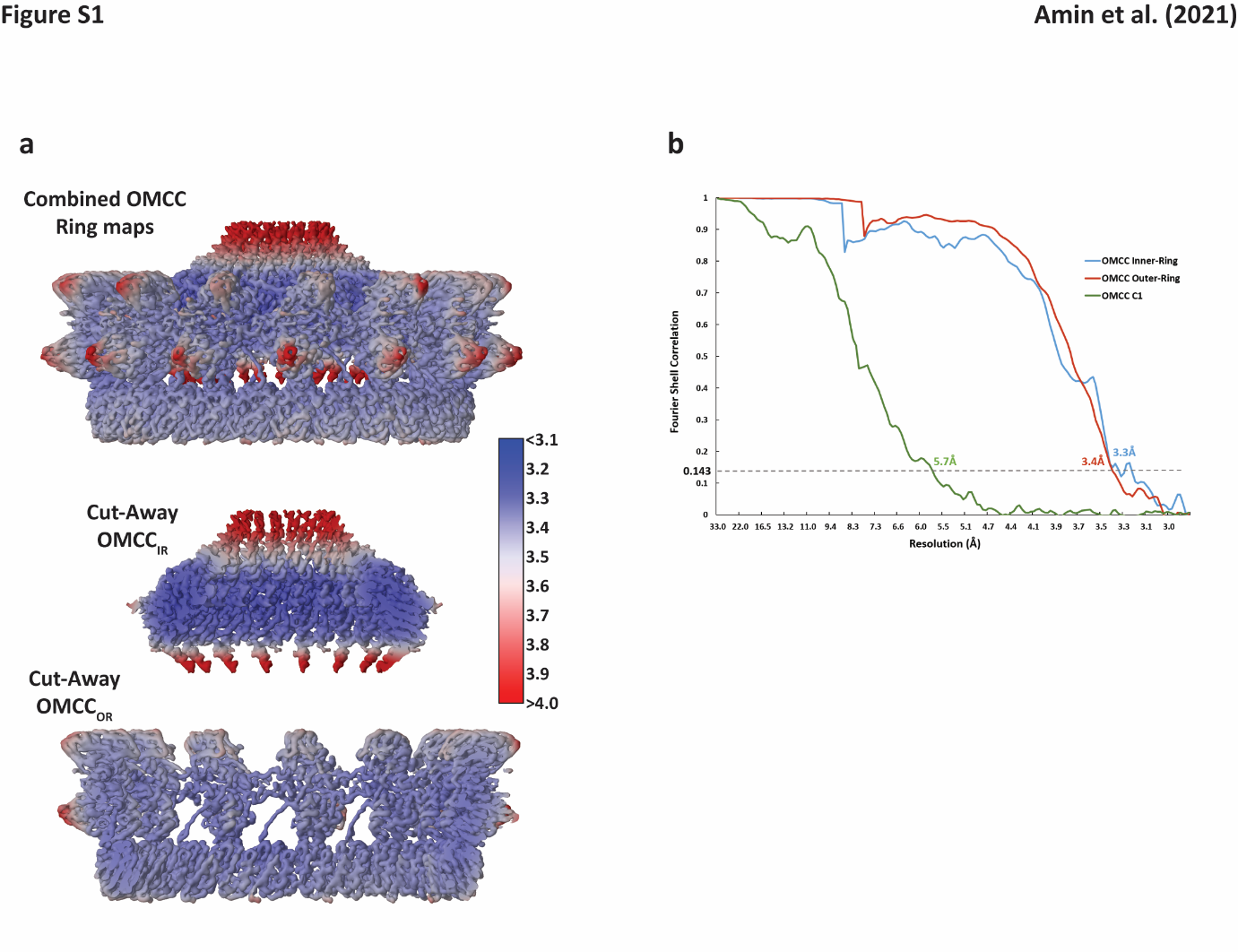
**

**Supplementary Figure 1: Resolution estimation of the OMCC_IR_ and OMCC_OR_ maps.**

a. Local resolution estimation of the combined (top panel) and individual OMCC_IR_ and OMCC_OR_ maps obtained using Relion 3.1. Colour code indicates that both maps local resolutions are ranging predominantly between 3.1 and 4.0 Å.

b. Overall resolution of the OMCC_C1_, OMCC_IR_ and OMCC_OR_ maps derived from two independently refined half-maps using the FSC=0.143 criteria. The curves represent the FSC of the OMCC_C1_ (green), OMCC_IR_ (blue) and OMCC_OR_ (red) mask-corrected maps.

**
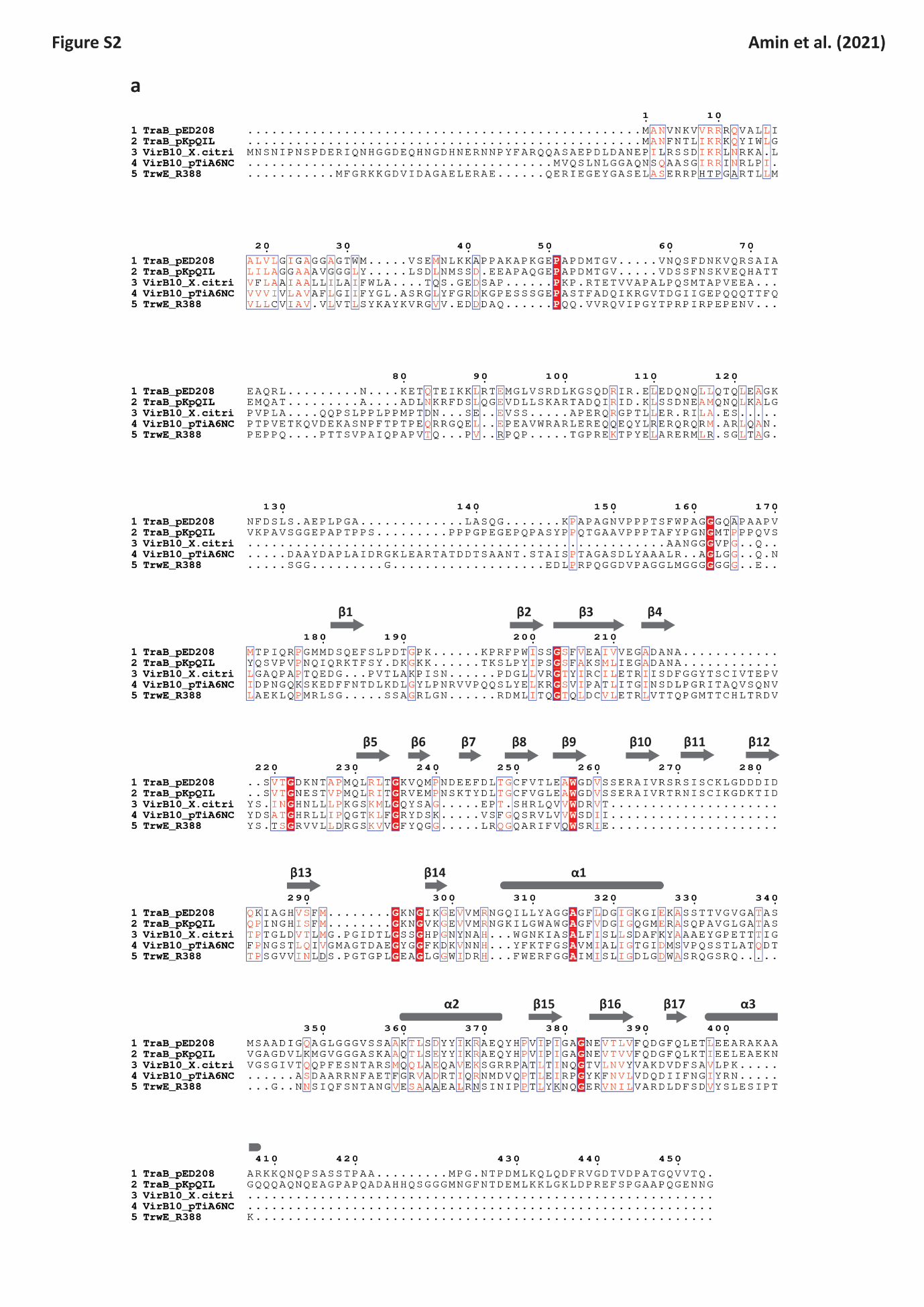
**

**
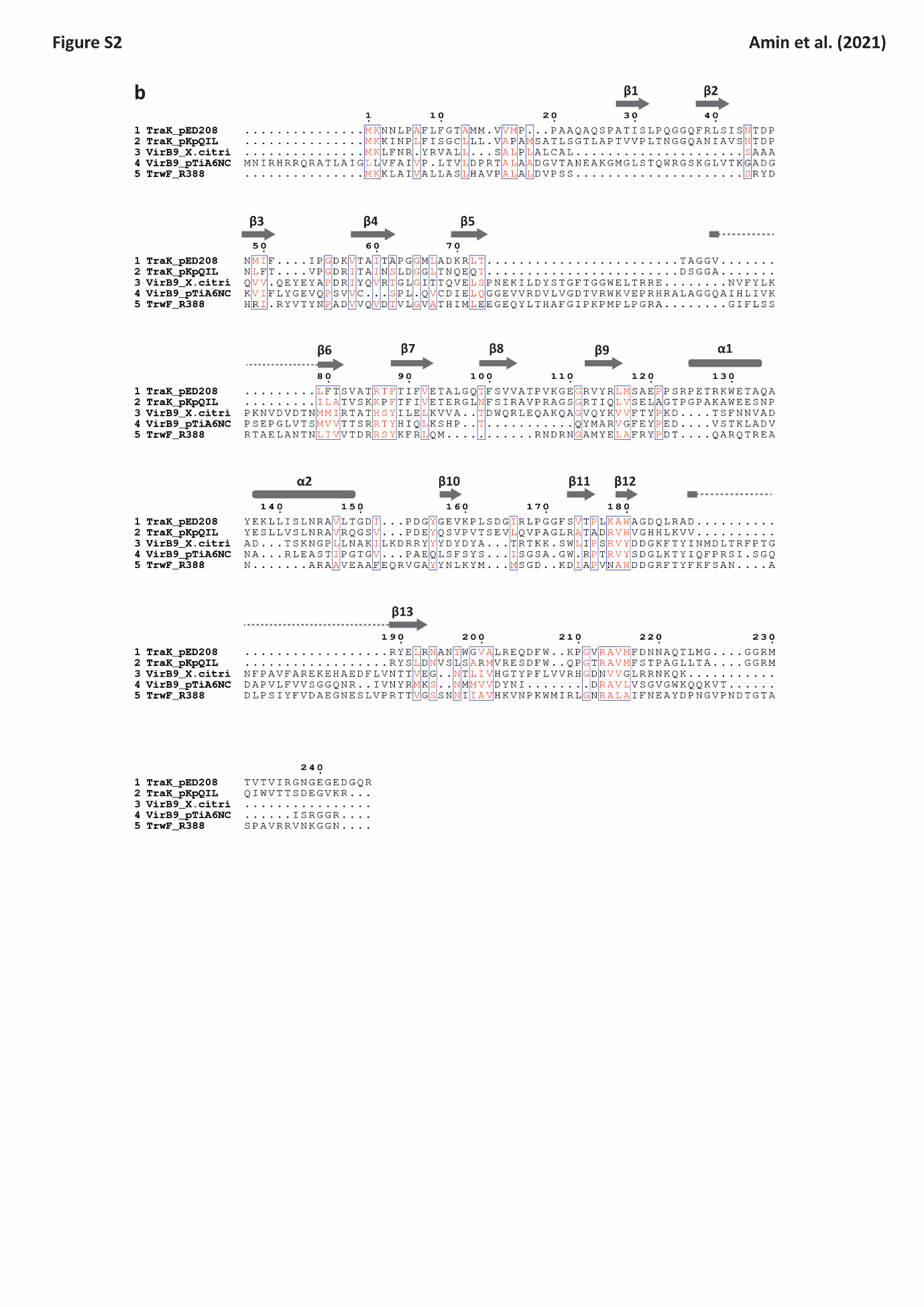
**

**
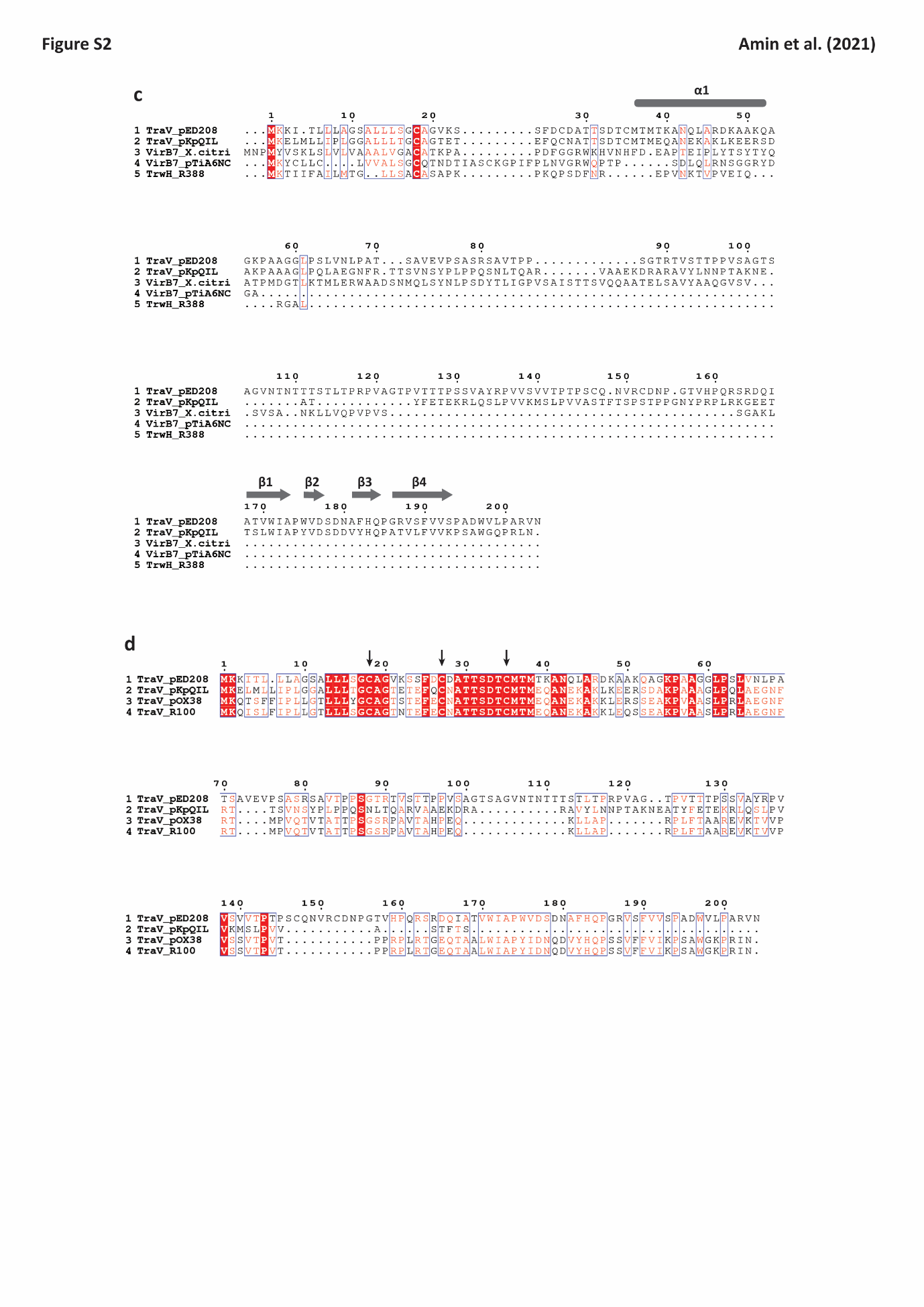
**

**
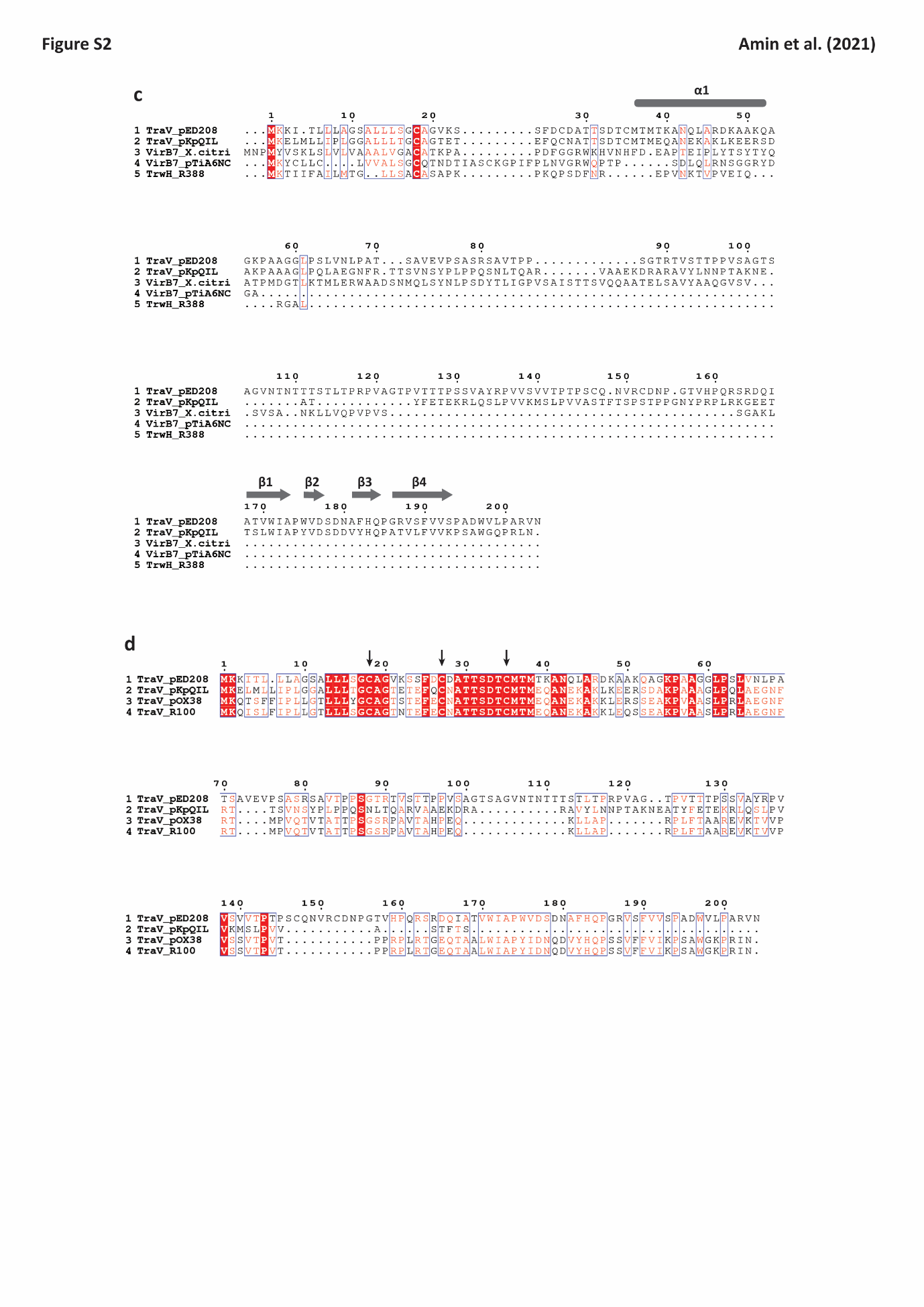
**

**Supplementary Figure 2: Protein sequence alignment of TraB, TraK and TraV with other T4SS protein homologues.**

a. Protein sequence alignment of *E. coli*_pED208_ TraB, *K. pneumoniae*_pKpQIL_ TraB, *X. citri* VirB10, *A. tumefaciens*_pTiA6NC_ VirB10 and *E. coli*_R388_ TrwE.

b. Protein sequence alignment of *E. coli*_pED208_ TraK, *K. pneumoniae*_pKpQIL_ TraK, *X. citri* VirB9, *A. tumefaciens*_pTiA6NC_ VirB9 and *E. coli*_R388_ TrwF.

c. Protein sequence alignment of *E. coli*_pED208_ TraV, *K. pneumoniae*_pKpQIL_ TraV, *X. citri* VirB9, *A. tumefaciens*_pTiA6NC_ VirB7 and *E. coli*_R388_ TrwH. The secondary structural features of TraB, TraK and TraV derived from the OMCC_IR_ and OMCC_OR_ models are depicted above the protein sequences.

d. Alignment of TraV proteins sequences from different members of the F-plasmid family. Black arrows indicate conservation of Cys18, Cys27 and Cys35 among the sequences.

**
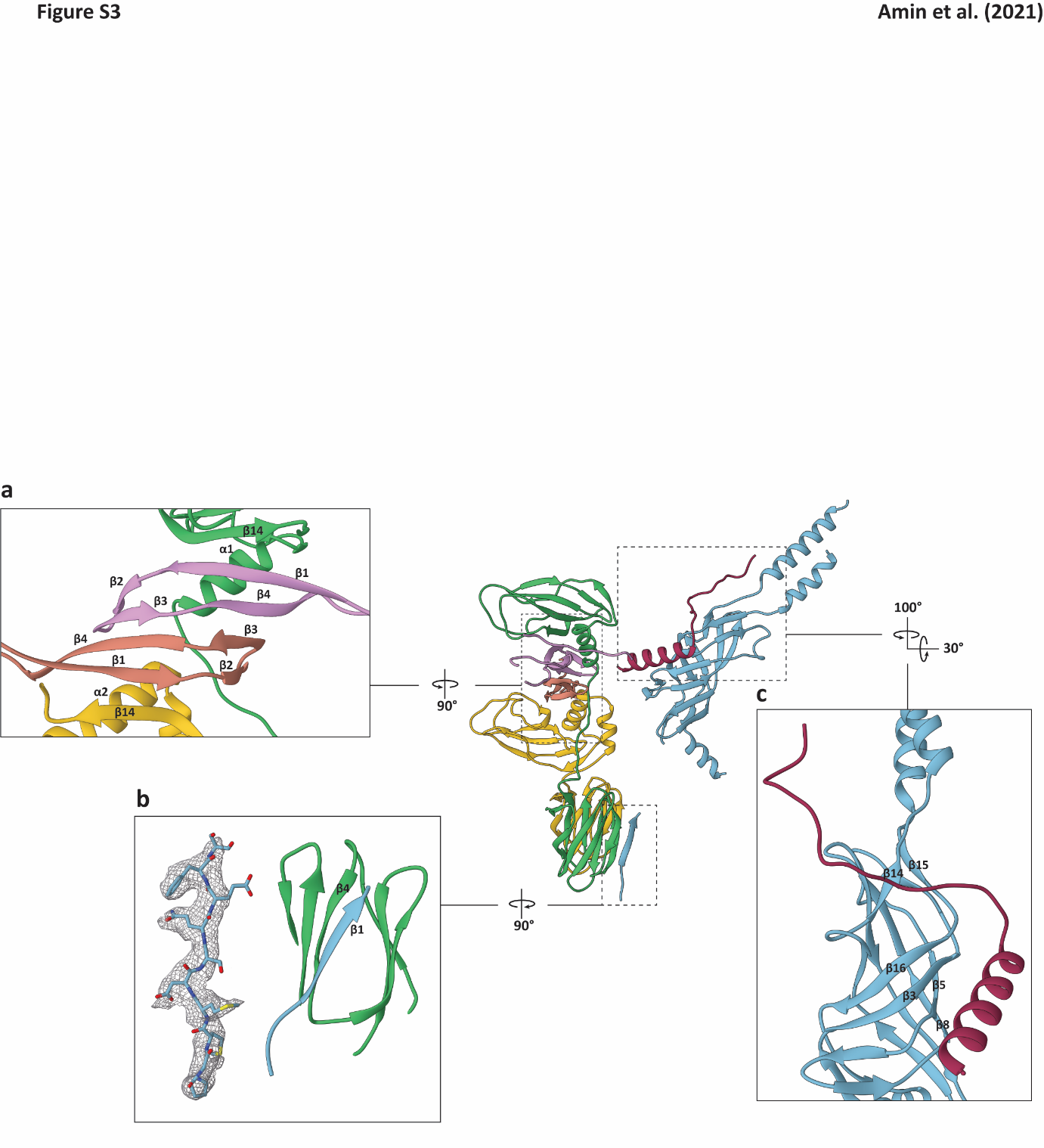
**

**Supplementary Figure 3: Interaction network within the ASU_IR_ and ASU_OR_.**

a. The interactions between the two antiparallel CTDs of TraV1 (pink) and TraV2 (orange) with TraK1_CTD_ (green) and TraK2_CTD_ (yellow) are established by large hydrophobic contacts mediated by TraV1_CTD_ β1-β4, with TraK1_CTD_ β14 and α1, and TraV2_CTD_ β1-β4, with TraK2_CTD_ β14 and α2.

b. TraB β1 (residues 177 – 186) at the I-layer folds into a β strand and is stabilized exclusively by main chain contacts with the TraK1 β4 strand. Details of the electron density map with the TraB β1 strand residues built in it.

c. TraV_NTD_ establish contacts through its α1 helix with TraB β3, β5, β8 and β16 strands and through its NTD loop with TraB β14 and β15 strands. Details of the residues within each interacting surface can be found in Supplementary Table 1.

**
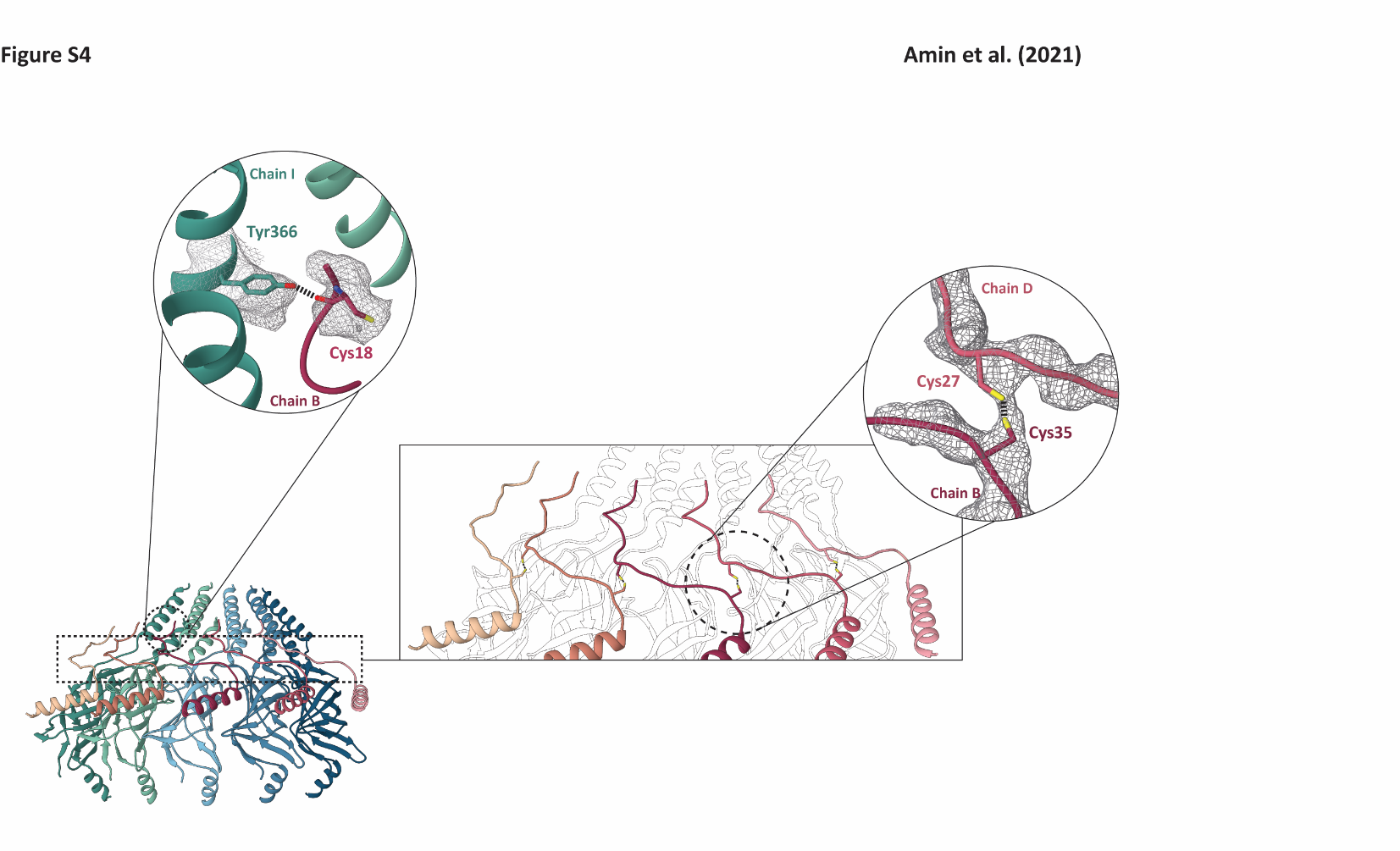
**

**Supplementary Figure 4: Inter ASU contacts within the OMCC_IR_ and OMCC_OR_**

The conserved TraV Cys18 interacts through its main chain oxygen with TraB Tyr366 side chain (inset, left). Details of the large network of TraV_NTD_ di-sulphide bond interactions between residues Cys35 and Cys27 that stabilise a wide TraV mesh that spreads over the OMCC_IR_ (inset, right). Details of the electron density from the interacting residues side chains are shown in mesh.

**
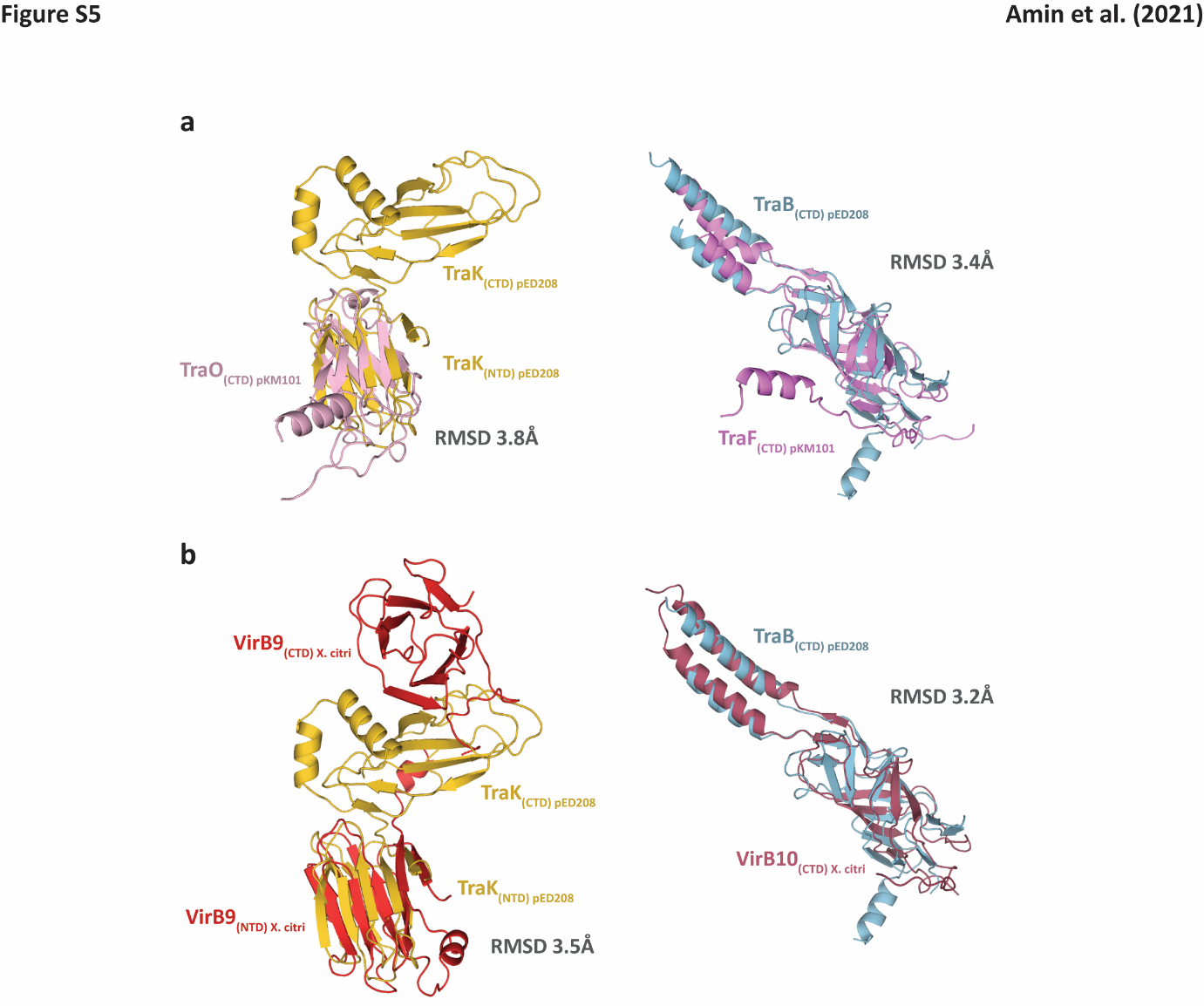
**

**Supplementary Figure 5: Similarities between TraB, TraK with their closest structural homologues.**

a. Structural alignment of the structure of TraK (yellow) and TraB (blue) with their pKM101 counterparts TraO (light pink) and TraF (pink).

b. Structural alignment of the structure of TraK (yellow) and TraB (blue) with their X. citri counterparts VirB9 (red) and VirB10 (dark red).

No structural homology was observed between the TraV structure and its closest homologues.

**Supplementary Table 1: Details of the interacting residues within the asymmetric unit in the inner and outer ring.**

**Interacting between Inner ring chain A and chain B Average interface area – 840.2 Å^2^**

| **Hydrogen bonds** | | Dist |
| --- | --- | --- |
| A:ASN 383[ ND2] | B:SER  32[ O  ] | 3.16 |
| A:ARG 232[ NH1] | B:ASN  42[ O  ] | 2.88 |
| A:LYS 298[ O  ] | B:THR  31[ N  ] | 3.50 |
| A:LYS 298[ O  ] | B:THR  31[ OG1] | 2.74 |
| A:GLU 384[ OE2] | B:LYS  48[ NZ ] | 3.32 |
| **Salt bridges** | |  |
| A:GLU 384[ OE2] | B:LYS  48[ NZ ] | 3.32 |

Interacting surface residues between two chains

| Chain A | Chain B |
| --- | --- |
| A:GLU 207 | B:PHE  25 |
| A:ILE 209 | B:CYS  27 |
| A:VAL 210 | B:ASP  28 |
| A:VAL 211 | B:ALA  29 |
| A:GLU 212 | B:THR  30 |
| A:GLN 230 | B:THR  31 |
| A:ARG 232 | B:SER  32 |
| A:THR 234 | B:ASP  33 |
| A:GLY 235 | B:THR  34 |
| A:LYS 236 | B:MET  36 |
| A:THR 248 | B:THR  37 |
| A:PHE 251 | B:MET  38 |
| A:SER 261 | B:ALA  41 |
| A:SER 262 | B:ASN  42 |
| A:GLU 263 | B:LEU  44 |
| A:PHE 291 | B:ALA  45 |
| A:MET 292 | B:ARG  46 |
| A:LYS 294 | B:LYS  48 |
| A:ILE 297 | B:ALA  49 |
| A:LYS 298 | B:GLN  52 |
| A:GLY 299 |  |
| A:GLU 300 |  |
| A:PRO 378 |  |
| A:ILE 379 |  |
| A:GLY 380 |  |
| A:ALA 381 |  |
| A:GLY 382 |  |
| A:ASN 383 |  |
| A:GLU 384 |  |

**Interacting between Outer ring chain A and chain B Average interface area - 1112.9 Å^2^**

| **Hydrogen bonds** | | Dist |
| --- | --- | --- |
| A:GLN  37[ O  ] | B:ALA  24[ N  ] | 3.18 |
| A:GLN  37[ O  ] | B:GLN  25[ N  ] | 3.01 |
| A:THR  61[ OG1] | B:ARG  71[ NH2] | 3.88 |
| A:GLU  93[ OE1] | B:ARG  71[ NH2] | 2.53 |
| A:THR  89[ OG1] | B:GLY 111[ N  ] | 3.83 |
| A:GLN  37[ N  ] | B:GLN  25[ O  ] | 3.34 |
| A:ARG  39[ NH1] | B:GLN  25[ OE1] | 3.85 |
| A:THR  89[ N  ] | B:ASP  46[ OD1] | 3.32 |
| A:ARG  39[ NH2] | B:GLU 110[ OE2] | 2.72 |
| A:ARG 213[ NH1] | B:GLU 131[ OE1] | 2.82 |
| A:TYR 136[ N  ] | B:THR 132[ O  ] | 3.69 |
| **Salt bridges** | |  |
| A:GLU  93[ OE1] | B:ARG  71[ NH2] | 2.53 |
| A:GLU  93[ OE2] | B:ARG  71[ NH2] | 2.75 |
| A:ARG  39[ NH2] | B:GLU 110[ OE1] | 3.77 |
| A:ARG  39[ NH1] | B:GLU 110[ OE2] | 3.92 |
| A:ARG  39[ NH2] | B:GLU 110[ OE2] | 2.72 |
| A:ARG 213[ NH1] | B:GLU 131[ OE1] | 2.82 |
| A:ARG 213[ NH2] | B:GLU 131[ OE1] | 3.50 |

Interacting surface residues between two chains

| Chain A | Chain B |
| --- | --- |
| A:ILE  30 | B:ALA  24 |
| A:SER  31 | B:GLN  25 |
| A:LEU  32 | B:SER  26 |
| A:PRO  33 | B:PRO  27 |
| A:GLN  34 | B:THR  45 |
| A:GLY  35 | B:ASP  46 |
| A:GLY  36 | B:PRO  47 |
| A:GLN  37 | B:MET  49 |
| A:PHE  38 | B:PHE  51 |
| A:ARG  39 | B:ASP  69 |
| A:THR  58 | B:ARG  71 |
| A:ALA  59 | B:THR  73 |
| A:THR  61 | B:THR  74 |
| A:PRO  63 | B:ALA  75 |
| A:GLY  64 | B:LEU  79 |
| A:ARG  86 | B:THR  81 |
| A:THR  87 | B:GLU 110 |
| A:PHE  88 | B:GLY 111 |
| A:THR  89 | B:ARG 112 |
| A:PHE  91 | B:VAL 113 |
| A:GLU  93 | B:ARG 115 |
| A:THR  94 | B:ARG 128 |
| A:ALA  95 | B:TRP 130 |
| A:GLY  97 | B:GLU 131 |
| A:GLN  98 | B:THR 132 |
| A:THR  99 | B:ALA 133 |
| A:SER 101 | B:GLN 134 |
| A:VAL 103 | B:GLU 137 |
| A:GLU 120 |  |
| A:ARG 128 |  |
| A:GLU 131 |  |
| A:THR 132 |  |
| A:GLN 134 |  |
| A:ALA 135 |  |
| A:TYR 136 |  |
| A:GLU 137 |  |
| A:LYS 138 |  |
| A:LEU 139 |  |
| A:GLN 184 |  |
| A:LEU 185 |  |
| A:ARG 213 |  |

**Interacting between Outer ring chain A and chain C Average interface area – 76.5 Å^2^**

| **Hydrogen bonds** | | Dist |
| --- | --- | --- |
| A:GLU 137[ OE1] | C:HIS 183[ ND1] | 3.89 |
| **Salt bridges** | |  |
| A:GLU 137[ OE1] | C:HIS 183[ ND1] | 3.89 |

Interacting surface residues between two chains

| Chain A | Chain C |
| --- | --- |
| A:GLU 137 | C:TRP 175 |
| A:LYS 138 | C:ASP 177 |
| A:ILE 141 | C:ALA 181 |
|  | C:HIS 183 |
|  | C:PRO 185 |

**Interacting between Outer ring chain A and chain D Average interface area – 697.9 Å^2^**

| **Hydrogen bonds** | | Dist |
| --- | --- | --- |
| A:GLU 204[ OE2] | D:THR 169[ OG1] | 3.78 |
| A:VAL 215[ O  ] | D:TRP 171[ N  ] | 3.06 |
| A:ARG 213[ O  ] | D:ALA 173[ N  ] | 2.92 |
| A:ASN 220[ ND2] | D:GLN 166[ OE1] | 3.35 |
| A:ASN 220[ ND2] | D:ILE 167[ O  ] | 2.52 |
| A:VAL 215[ N  ] | D:TRP 171[ O  ] | 3.14 |
| A:ARG 145[ NH1] | D:VAL 191[ O  ] | 3.80 |
| A:ARG 145[ NH2] | D:VAL 191[ O  ] | 3.64 |
| **Salt bridges** | |  |
| A:GLU 204[ OE1] | D:ARG 187[ NE ] | 3.84 |
| A:GLU 204[ OE1] | D:ARG 187[ NH1] | 3.66 |

Interacting surface residues between two chains

| Chain A | Chain D |
| --- | --- |
| A:TYR 136 | D:GLN 166 |
| A:GLU 137 | D:ILE 167 |
| A:LEU 140 | D:ALA 168 |
| A:ILE 141 | D:THR 169 |
| A:ASN 144 | D:VAL 170 |
| A:ARG 145 | D:TRP 171 |
| A:LEU 148 | D:ILE 172 |
| A:THR 149 | D:ALA 173 |
| A:GLU 204 | D:PRO 174 |
| A:GLN 205 | D:TRP 175 |
| A:TRP 208 | D:ARG 187 |
| A:VAL 212 | D:VAL 188 |
| A:ARG 213 | D:PHE 190 |
| A:ALA 214 | D:VAL 191 |
| A:VAL 215 | D:VAL 192 |
| A:MET 216 |  |
| A:PHE 217 |  |
| A:ASP 218 |  |
| A:ASN 220 |  |

**Interacting between Outer ring chain B and chain C Interface area – 746.3 Å^2^**

| **Hydrogen bonds** | | Dist |
| --- | --- | --- |
| B:VAL 215[ O  ] | C:TRP 171[ N  ] | 3.43 |
| B:ARG 213[ O  ] | C:ALA 173[ N  ] | 3.28 |
| B:GLU 137[ OE1] | C:TRP 175[ NE1] | 2.54 |
| B:GLU 204[ OE2] | C:ARG 187[ NE ] | 2.65 |
| B:VAL 215[ N  ] | C:TRP 171[ O  ] | 3.41 |
| B:ARG 145[ NH1] | C:VAL 191[ O  ] | 2.32 |
| **Salt bridges** | |  |
| B:GLU 204[ OE2] | C:ARG 187[ NE ] | 2.65 |
| B:GLU 204[ OE2] | C:ARG 187[ NH1] | 3.30 |

Interacting surface residues between two chains

| Chain B | Chain C |
| --- | --- |
| B:GLN 134 | C:GLN 166 |
| B:TYR 136 | C:ILE 167 |
| B:GLU 137 | C:ALA 168 |
| B:LEU 140 | C:THR 169 |
| B:ILE 141 | C:VAL 170 |
| B:ASN 144 | C:TRP 171 |
| B:ARG 145 | C:ILE 172 |
| B:LEU 148 | C:ALA 173 |
| B:THR 149 | C:PRO 174 |
| B:GLU 204 | C:TRP 175 |
| B:GLN 205 | C:ASP 177 |
| B:TRP 208 | C:HIS 183 |
| B:VAL 212 | C:PRO 185 |
| B:ARG 213 | C:ARG 187 |
| B:ALA 214 | C:VAL 188 |
| B:VAL 215 | C:PHE 190 |
| B:MET 216 | C:VAL 191 |
| B:PHE 217 | C:VAL 192 |
| B:ASP 218 |  |
| B:ASN 220 |  |
| B:GLN 222 |  |

**Interacting between Outer ring chain B and chain D Interface area – 261.7 Å^2^**

| **Hydrogen bonds** | | Dist |
| --- | --- | --- |
| B:GLU 131[ O  ] | D:SER 178[ N  ] | 3.26 |
| B:LYS 129[ O  ] | D:SER 178[ OG ] | 2.81 |
| B:ALA 133[ N  ] | D:ASP 177[ OD1] | 3.47 |

Interacting surface residues between two chains

| Chain B | Chain D |
| --- | --- |
| B:LYS 129 | D:TRP 175 |
| B:TRP 130 | D:VAL 176 |
| B:GLU 131 | D:ASP 177 |
| B:THR 132 | D:SER 178 |
| B:ALA 133 | D:ASP 179 |
| B:GLU 137 | D:ASN 180 |
| B:LYS 138 | D:ALA 181 |
| B:ILE 141 | D:HIS 183 |
| B:ARG 145 |  |

**Interacting between Outer ring chain B and chain E Interface area – 404.3 Å^2^**

| **Hydrogen bonds** | | Dist |
| --- | --- | --- |
| B:THR  61[ OG1] | E:SER 182[ OG ] | 3.54 |
| B:ILE  60[ O  ] | E:GLN 183[ N  ] | 3.44 |
| B:THR  58[ O  ] | E:PHE 185[ N  ] | 2.75 |
| B:ALA  62[ N  ] | E:ASP 181[ O  ] | 3.57 |
| B:ILE  60[ N  ] | E:GLN 183[ O  ] | 3.37 |

Interacting surface residues between two chains

| Chain B | Chain E |
| --- | --- |
| B:VAL  57 | E:MET 179 |
| B:THR  58 | E:MET 180 |
| B:ALA  59 | E:ASP 181 |
| B:ILE  60 | E:SER 182 |
| B:THR  61 | E:GLN 183 |
| B:ALA  62 | E:GLU 184 |
| B:PRO  63 | E:PHE 185 |
| B:MET  66 | E:SER 186 |
| B:LEU  67 |  |
| B:LYS  70 |  |
| B:ARG  71 |  |
| B:LEU  72 |  |
| B:VAL  78 |  |

**Interacting between Outer ring chain C and chain D Interface area – 404.7 Å^2^**

| **Hydrogen bonds** | | |
| --- | --- | --- |
| C:PHE 182[ O  ] | D:SER 189[ N  ] | 2.52 |
| C:GLN 184[ O  ] | D:ARG 187[ N  ] | 3.10 |
| C:ARG 187[ O  ] | D:GLN 184[ N  ] | 3.60 |
| C:SER 189[ O  ] | D:PHE 182[ N  ] | 2.90 |
| C:SER 189[ N  ] | D:PHE 182[ O  ] | 3.04 |
| C:GLN 184[ N  ] | D:ARG 187[ O  ] | 2.46 |
| C:PHE 182[ N  ] | D:SER 189[ O  ] | 2.73 |

Interacting surface residues between two chains

| Chain C | Chain D |
| --- | --- |
| C:THR 169 | D:ASP 179 |
| C:ASN 180 | D:ASN 180 |
| C:ALA 181 | D:ALA 181 |
| C:PHE 182 | D:PHE 182 |
| C:HIS 183 | D:HIS 183 |
| C:GLN 184 | D:GLN 184 |
| C:PRO 185 | D:PRO 185 |
| C:GLY 186 | D:GLY 186 |
| C:ARG 187 | D:ARG 187 |
| C:VAL 188 | D:VAL 188 |
| C:SER 189 | D:SER 189 |
| C:PHE 190 | D:PHE 190 |
| C:VAL 191 | D:VAL 191 |

**Supplementary Table 2: Details of the interacting residues between asymmetric units in the inner and outer ring.**

**Interacting between Inner ring ASU and chain E ASU interacting surface area – 2024.1 Å^2^**

| Hydrogen bonds | | Dist |
| --- | --- | --- |
| A:SER 202[ N  ] | E:ASP 282[ OD2] | 3.44 |
| A:SER 202[ N  ] | E:ASP 282[ OD1] | 3.53 |
| A:ASN 241[ ND2] | E:THR 248[ O  ] | 3.80 |
| A:ASN 241[ ND2] | E:GLY 249[ O  ] | 3.23 |
| A:HIS 288[ NE2] | E:GLU 212[ OE1] | 2.98 |
| A:ASN 295[ N  ] | E:GLY 213[ O  ] | 3.19 |
| A:ASN 295[ ND2] | E:PRO 228[ O  ] | 3.37 |
| A:TRP 199[ O  ] | E:LYS 275[ NZ ] | 2.52 |
| A:GLY 203[ O  ] | E:GLN 230[ NE2] | 3.26 |
| A:ASN 241[ OD1] | E:LYS 275[ N  ] | 2.15 |
| A:ASN 241[ OD1] | E:LEU 276[ N  ] | 2.98 |
| A:ASP 242[ OD1] | E:ARG 232[ NH2] | 3.34 |
| A:GLU 263[ OE2] | E:ARG 304[ NH1] | 2.64 |
| A:GLY 293[ O  ] | E:GLY 213[ N  ] | 2.72 |
| A:ASN 305[ OD1] | E:TYR 365[ OH ] | 3.06 |
| A:ALA 370[ O  ] | E:TYR 365[ OH ] | 3.78 |
| A:GLN 390[ OE1] | E:ARG 270[ NH1] | 3.65 |
| A:ASP 391[ OD1] | E:ARG 270[ NH1] | 3.34 |
| B:LYS  22[ O  ] | E:MET 303[ N  ] | 3.25 |
| B:SER  23[ OG ] | E:ARG 304[ NE ] | 3.79 |
| B:LYS  22[ N  ] | E:VAL 301[ O  ] | 3.75 |
| Salt bridges | |  |
| A:HIS 288[ NE2] | E:GLU 212[ OE1] | 2.98 |
| A:LYS 294[ NZ ] | E:ASP 215[ OD1] | 3.65 |
| A:ASP 242[ OD1] | E:ARG 232[ NH2] | 3.34 |
| A:GLU 263[ OE1] | E:ARG 304[ NH1] | 3.52 |
| A:GLU 263[ OE2] | E:ARG 304[ NH1] | 2.64 |
| A:GLU 263[ OE2] | E:ARG 304[ NH2] | 3.81 |
| A:ASP 391[ OD1] | E:ARG 270[ NH1] | 3.34 |
| A:ASP 391[ OD2] | E:ARG 270[ NH1] | 3.57 |
| B:LYS  22[ NZ ] | E:GLU 300[ OE1] | 3.89 |

Interacting surface residues between Inner ring ASU and chain E

| Chain A | Chain E |
| --- | --- |
| A:TRP 199 | E:VAL 211 |
| A:SER 201 | E:GLU 212 |
| A:SER 202 | E:GLY 213 |
| A:GLY 203 | E:ALA 214 |
| A:SER 204 | E:ASP 215 |
| A:PHE 205 | E:ALA 227 |
| A:SER 219 | E:PRO 228 |
| A:VAL 220 | E:MET 229 |
| A:GLN 238 | E:GLN 230 |
| A:MET 239 | E:ARG 232 |
| A:PRO 240 | E:THR 248 |
| A:ASN 241 | E:GLY 249 |
| A:ASP 242 | E:CYS 250 |
| A:GLU 243 | E:PHE 251 |
| A:ASP 259 | E:THR 253 |
| A:VAL 260 | E:ARG 270 |
| A:SER 261 | E:SER 271 |
| A:SER 262 | E:ILE 272 |
| A:GLU 263 | E:SER 273 |
| A:ARG 264 | E:CYS 274 |
| A:HIS 288 | E:LYS 275 |
| A:SER 290 | E:LEU 276 |
| A:GLY 293 | E:GLY 277 |
| A:LYS 294 | E:ASP 280 |
| A:ASN 295 | E:ILE 281 |
| A:LYS 298 | E:ASP 282 |
| A:VAL 301 | E:LYS 284 |
| A:MET 303 | E:MET 303 |
| A:ASN 305 | E:ARG 304 |
| A:LEU 309 | E:LEU 318 |
| A:ALA 312 | E:GLU 326 |
| A:GLY 313 | E:LYS 327 |
| A:GLY 316 | E:ALA 359 |
| A:PHE 317 | E:THR 361 |
| A:GLY 320 | E:LEU 362 |
| A:GLY 324 | E:TYR 365 |
| A:ILE 325 | E:TYR 366 |
| A:LYS 327 | E:LYS 368 |
| A:SER 363 | E:ARG 369 |
| A:ASP 364 | E:GLN 372 |
| A:ILE 367 | E:TYR 373 |
| A:ALA 370 | E:HIS 374 |
| A:GLU 371 | E:VAL 376 |
| A:PRO 375 | E:PRO 378 |
| A:VAL 388 | E:ILE 379 |
| A:GLN 390 | E:GLY 380 |
| A:ASP 391 | E:ALA 381 |
| Chain B | E:LEU 398 |
| B:GLY  17 | E:VAL 260 |
| B:CYS  18 | E:GLU 263 |
| B:ALA  19 | E:GLU 300 |
| B:GLY  20 | E:VAL 301 |
| B:VAL  21 | E:VAL 302 |
| B:LYS  22 | E:ASN 305 |
| B:SER  23 | E:GLY 306 |
| B:SER  24 | E:LEU 309 |
| B:PHE  25 | E:LEU 310 |
|  | E:GLY 313 |
|  | E:PRO 375 |
|  | Chain E |
|  | E:VAL 211 |
|  | E:GLU 212 |
|  | E:GLY 213 |
|  | E:ALA 214 |
|  | E:ASP 215 |

**Interacting between Inner ring ASU and chain C ASU interacting surface area – 1894.1 Å^2^**

| Hydrogen bonds | | Dist |
| --- | --- | --- |
| A:GLY 213[ N  ] | C:GLY 293[ O  ] | 3.00 |
| A:GLN 230[ NE2] | C:GLY 203[ O  ] | 2.88 |
| A:ARG 232[ NH2] | C:PRO 240[ O  ] | 3.65 |
| A:ARG 232[ NH2] | C:ASP 242[ OD1] | 2.92 |
| A:CYS 250[ SG ] | C:ASN 241[ OD1] | 3.83 |
| A:ARG 270[ NH1] | C:ASP 391[ OD2] | 3.23 |
| A:SER 273[ OG ] | C:SER 202[ O  ] | 2.99 |
| A:LYS 275[ N  ] | C:ASN 241[ OD1] | 2.64 |
| A:LYS 275[ NZ ] | C:TRP 199[ O  ] | 2.37 |
| A:LEU 276[ N  ] | C:ASN 241[ OD1] | 3.76 |
| A:ARG 304[ NH1] | C:GLU 263[ OE2] | 2.71 |
| A:TYR 365[ OH ] | C:ALA 370[ O  ] | 3.74 |
| A:TYR 365[ OH ] | C:ASN 305[ OD1] | 3.27 |
| A:GLU 212[ OE1] | C:HIS 288[ NE2] | 2.78 |
| A:GLY 213[ O  ] | C:ASN 295[ N  ] | 3.63 |
| A:PRO 228[ O  ] | C:ASN 295[ ND2] | 3.78 |
| A:THR 248[ O  ] | C:ASN 241[ ND2] | 3.56 |
| A:GLY 249[ O  ] | C:ASN 241[ ND2] | 2.61 |
| A:ASP 282[ OD2] | C:SER 202[ OG ] | 3.62 |
| Salt bridges | |  |
| A:ARG 232[ NH2] | C:ASP 242[ OD1] | 2.92 |
| A:ARG 270[ NH1] | C:ASP 391[ OD2] | 3.23 |
| A:ARG 270[ NH1] | C:ASP 391[ OD1] | 3.51 |
| A:ARG 304[ NH1] | C:GLU 263[ OE1] | 3.39 |
| A:ARG 304[ NH1] | C:GLU 263[ OE2] | 2.71 |
| A:ARG 304[ NH2] | C:GLU 263[ OE2] | 3.37 |
| A:GLU 212[ OE1] | C:HIS 288[ NE2] | 2.78 |
| A:GLU 212[ OE2] | C:HIS 288[ NE2] | 3.58 |
| B:ASP  33[ OD2] | C:LYS 294[ NZ ] | 3.90 |
| B:ARG  46[ NE ] | C:ASP 242[ OD2] | 3.64 |

Interacting surface residues between Inner ring ASU and chain C

| Chain A | Chain C |
| --- | --- |
| A:VAL 211 | C:PRO 198 |
| A:GLU 212 | C:TRP 199 |
| A:GLY 213 | C:SER 201 |
| A:ALA 214 | C:SER 202 |
| A:ASP 215 | C:GLY 203 |
| A:ALA 227 | C:SER 204 |
| A:PRO 228 | C:PHE 205 |
| A:GLN 230 | C:SER 219 |
| A:ARG 232 | C:VAL 220 |
| A:THR 248 | C:GLN 238 |
| A:GLY 249 | C:MET 239 |
| A:CYS 250 | C:PRO 240 |
| A:PHE 251 | C:ASN 241 |
| A:THR 253 | C:ASP 242 |
| A:ARG 270 | C:GLU 243 |
| A:SER 271 | C:ASP 259 |
| A:ILE 272 | C:VAL 260 |
| A:SER 273 | C:SER 261 |
| A:CYS 274 | C:SER 262 |
| A:LYS 275 | C:GLU 263 |
| A:LEU 276 | C:ARG 264 |
| A:GLY 277 | C:HIS 288 |
| A:ASP 280 | C:SER 290 |
| A:ILE 281 | C:GLY 293 |
| A:ASP 282 | C:LYS 294 |
| A:MET 303 | C:ASN 295 |
| A:ARG 304 | C:LYS 298 |
| A:LEU 318 | C:VAL 301 |
| A:GLU 326 | C:MET 303 |
| A:LYS 327 | C:ASN 305 |
| A:ALA 359 | C:LEU 309 |
| A:THR 361 | C:ALA 312 |
| A:LEU 362 | C:GLY 313 |
| A:TYR 365 | C:GLY 316 |
| A:TYR 366 | C:PHE 317 |
| A:LYS 368 | C:GLY 324 |
| A:ARG 369 | C:ILE 325 |
| A:GLN 372 | C:LYS 327 |
| A:TYR 373 | C:SER 363 |
| A:HIS 374 | C:ASP 364 |
| A:VAL 376 | C:ILE 367 |
| A:PRO 378 | C:ALA 370 |
| A:ILE 379 | C:GLU 371 |
| A:GLY 380 | C:PRO 375 |
| A:ALA 381 | C:VAL 388 |
| A:LEU 398 | C:GLN 390 |
| Chain B | C:ASP 391 |
| B:ASP  33 | C:GLU 207 |
| B:CYS  35 | C:MET 292 |
| B:MET  36 | C:THR 386 |
| B:THR  37 |  |
| B:MET  38 |  |
| B:THR  39 |  |
| B:ASN  42 |  |
| B:ARG  46 |  |
|  |  |

**Interacting between Inner ring ASU and chain D ASU interacting surface area – 541.2 Å^2^**

| Disulfide bonds | | Dist |
| --- | --- | --- |
| B:CYS  35[ SG ] | D:CYS  27[ SG ] | 2.03 |
| Hydrogen bonds | |  |
| A:MET 303[ N  ] | D:LYS  22[ O  ] | 3.53 |
| A:MET 303[ O  ] | D:SER  23[ OG ] | 2.58 |

Interacting surface residues between Inner ring ASU and chain D

| Chain A | Chain D |
| --- | --- |
| A:ASP 215 | D:GLY  17 |
| A:GLU 263 | D:CYS  18 |
| A:GLU 300 | D:ALA  19 |
| A:VAL 301 | D:GLY  20 |
| A:VAL 302 | D:VAL  21 |
| A:MET 303 | D:LYS  22 |
| A:ARG 304 | D:SER  23 |
| A:ASN 305 | D:SER  24 |
| A:GLY 306 | D:PHE  25 |
| A:LEU 309 | D:ASP  26 |
| A:LEU 310 | D:CYS  27 |
| A:GLY 313 | D:ALA  29 |
| A:GLY 314 |  |
| A:PRO 375 |  |
| A:VAL 376 |  |
| A:PRO 378 |  |
| Chain B |  |
| B:THR  31 |  |
| B:ASP  33 |  |
| B:THR  34 |  |
| B:CYS  35 |  |

**Interacting between Inner ring ASU and chain F ASU interacting surface area – 457.3 Å^2^**

| Disulfide bonds | | |
| --- | --- | --- |
| B:CYS  27[ SG ] | F:CYS  35[ SG ] | 1.36 |

Interacting surface residues between Inner ring ASU and chain F

| Chain A | Chain F |
| --- | --- |
| A:PHE 205 | F:ASP  33 |
| A:GLU 207 | F:CYS  35 |
| A:GLN 238 | F:MET  36 |
| A:ASP 242 | F:THR  37 |
| A:HIS 288 | F:MET  38 |
| A:SER 290 | F:THR  39 |
| A:MET 292 | F:ASN  42 |
| A:GLY 293 | F:ARG  46 |
| A:LYS 294 | F:THR  34 |
| A:THR 386 | F:THR  31 |
| A:VAL 388 |  |
| Chain B |  |
| B:PHE  25 |  |
| B:ASP  26 |  |
| B:CYS  27 |  |
| B:ALA  29 |  |

**Interacting between Inner ring ASU and chain H ASU interacting surface area – 171.7 Å^2^**

| Hydrogen bonds | | Dist |
| --- | --- | --- |
| A:TYR 366[ OH ] | H:CYS  18[ O  ] | 2.39 |

Interacting surface residues between Inner ring ASU and chain H

| Chain A | Chain H |
| --- | --- |
| A:GLN 307 | H:GLY  17 |
| A:ILE 308 | H:CYS  18 |
| A:TYR 311 | H:ALA  19 |
| A:LEU 362 | H:GLY  20 |
| A:TYR 366 | H:VAL  21 |
| A:ARG 369 |  |
| A:TYR 373 |  |

**Interacting between Inner ring ASU and chain I ASU interacting surface area – 178.4 Å^2^**

| Hydrogen bonds | | Dist |
| --- | --- | --- |
| B:CYS  18[ O  ] | I:TYR 366[ OH ] | 2.10 |

Interacting surface residues between Inner ring ASU and chain I

| Chain B | Chain I |
| --- | --- |
| B:GLY  17 | I:GLN 307 |
| B:CYS  18 | I:ILE 308 |
| B:ALA  19 | I:TYR 311 |
| B:GLY  20 | I:LEU 362 |
| B:VAL  21 | I:TYR 366 |
|  | I:ARG 369 |
|  | I:TYR 373 |

**Interacting between Outer ring ASU and chain F ASU interacting surface area – 1009.9 Å^2^**

| Hydrogen bonds | | Dist |
| --- | --- | --- |
| B:THR  89[ N  ] | F:ASP  46[ OD1] | 3.63 |
| B:ARG  39[ NH2] | F:GLU 110[ OE1] | 2.87 |
| B:GLN  37[ NE2] | F:GLY 111[ O  ] | 2.53 |
| B:THR  61[ OG1] | F:ARG  71[ NH1] | 3.76 |
| B:GLU  93[ OE2] | F:ARG  71[ NH1] | 3.35 |
| B:THR  89[ OG1] | F:GLY 111[ N  ] | 3.65 |
| E:MET 180[ N  ] | F:ASP  69[ OD2] | 3.55 |
| Salt bridges | |  |
| B:ARG  39[ NH2] | F:GLU 110[ OE1] | 2.87 |
| B:GLU  93[ OE2] | F:ARG  71[ NH1] | 3.35 |

Interacting surface residues between Outer ring ASU and chain F

| Chain B | Chain F |
| --- | --- |
| B:SER  31 | F:ALA  24 |
| B:LEU  32 | F:GLN  25 |
| B:PRO  33 | F:SER  26 |
| B:GLN  34 | F:PRO  27 |
| B:GLY  35 | F:THR  45 |
| B:GLY  36 | F:ASP  46 |
| B:GLN  37 | F:PRO  47 |
| B:PHE  38 | F:ASN  48 |
| B:ARG  39 | F:MET  49 |
| B:THR  58 | F:PHE  51 |
| B:ALA  59 | F:ARG  71 |
| B:ILE  60 | F:THR  73 |
| B:THR  61 | F:THR  74 |
| B:PRO  63 | F:ALA  75 |
| B:GLY  64 | F:LEU  79 |
| B:ARG  86 | F:GLU 110 |
| B:THR  87 | F:GLY 111 |
| B:PHE  88 | F:ARG 112 |
| B:THR  89 | F:VAL 113 |
| B:PHE  91 | F:ARG 115 |
| B:GLU  93 | F:LYS 178 |
| B:THR  94 | F:ALA  68 |
| B:GLY  97 | F:ASP  69 |
| B:THR  99 | F:LYS  70 |
| B:PHE 100 | F:PHE  80 |
| B:SER 101 | F:THR  81 |
| B:VAL 103 | F:VAL  83 |
| B:GLU 120 | F:GLU 204 |
| Chain E | F:ASN 220 |
| E:PRO 177 | F:ALA 221 |
| E:GLY 178 | F:GLN 222 |
| E:MET 179 |  |
| E:MET 180 |  |
| E:SER 182 |  |
| E:GLN 183 |  |
| E:GLU 184 |  |
| Chain C |  |
| C:THR 157 |  |
| C:VAL 158 |  |
| C:HIS 159 |  |

**Interacting between Outer ring ASU and chain G ASU interacting surface area – 246.3 Å^2^**

| Hydrogen bonds | | Dist |
| --- | --- | --- |
| C:SER  71[ OG ] | G:GLN 184[ OE1] | 2.87 |
| C:ALA  72[ O  ] | G:ARG 213[ NH1] | 3.57 |
| C:GLU  74[ OE2] | G:TYR 136[ OH ] | 2.96 |
| C:GLU  74[ OE2] | G:ARG 213[ NH1] | 2.64 |
| Salt bridges | |  |
| C:GLU  74[ OE1] | G:ARG 213[ NE ] | 3.42 |
| C:GLU  74[ OE1] | G:ARG 213[ NH1] | 3.48 |
| C:GLU  74[ OE1] | G:ARG 213[ NH2] | 3.58 |
| C:GLU  74[ OE2] | G:ARG 213[ NE ] | 3.20 |
| C:GLU  74[ OE2] | G:ARG 213[ NH1] | 2.64 |

Interacting surface residues between Outer ring ASU and chain G

| Chain C | Chain G |
| --- | --- |
| C:THR  70 | G:TYR 136 |
| C:SER  71 | G:GLN 184 |
| C:ALA  72 | G:LEU 185 |
| C:GLU  74 | G:TRP 208 |
| C:VAL  75 | G:PRO 210 |
| C:PRO  76 | G:GLY 211 |
| C:SER  77 | G:VAL 212 |
| C:ALA  78 | G:ARG 213 |

**Interacting between Outer ring ASU and chain H ASU interacting surface area – 349.6 Å^2^**

| Hydrogen bonds | | Dist |
| --- | --- | --- |
| C:VAL  73[ O  ] | H:VAL 176[ N  ] | 3.13 |
| C:VAL  75[ N  ] | H:PRO 174[ O  ] | 3.43 |
| C:ARG 162[ NH2] | H:ASN 180[ OD1] | 3.81 |
| C:TRP 197[ NE1] | H:ASN 180[ OD1] | 2.69 |

Interacting surface residues between Outer ring ASU and chain H

| Chain C | Chain H |
| --- | --- |
| C:ALA  72 | H:ALA 173 |
| C:VAL  73 | H:PRO 174 |
| C:GLU  74 | H:TRP 175 |
| C:VAL  75 | H:VAL 176 |
| C:SER  77 | H:ASP 177 |
| C:VAL 158 | H:SER 178 |
| C:HIS 159 | H:ASN 180 |
| C:PRO 160 | H:ALA 181 |
| C:ARG 162 | H:PHE 182 |
| C:TRP 197 | H:GLN 184 |
| C:VAL 198 |  |
| C:LEU 199 |  |

**Interacting between Outer ring ASU and chain I ASU interacting surface area – 1065 Å^2^**

| Hydrogen bonds | | Dist |
| --- | --- | --- |
| C:ARG 162[ O  ] | I:ARG 164[ NH1] | 2.95 |
| C:ASP 165[ OD2] | I:ARG 162[ NH1] | 3.35 |
| Salt bridges | |  |
| C:ASP 165[ OD2] | I:ARG 162[ NH1] | 3.35 |

Interacting surface residues between Outer ring ASU and chain I

| Chain A | Chain I |
| --- | --- |
| A:ARG 167 | I:VAL 158 |
| A:ARG 203 | I:HIS 159 |
| A:GLN 205 | I:PRO 160 |
| A:TRP 208 | I:GLN 161 |
| A:PRO 210 | I:ARG 162 |
| Chain B | I:SER 163 |
| B:LYS 129 | I:ARG 164 |
| B:GLN 222 | I:ASP 165 |
| Chain C | I:ILE 167 |
| C:THR 157 | I:THR 169 |
| C:HIS 159 | I:ARG 187 |
| C:PRO 160 | I:SER 189 |
| C:GLN 161 | I:PHE 190 |
| C:SER 163 | I:VAL 191 |
| C:ARG 164 | I:ALA 195 |
| C:ASP 165 | I:TRP 197 |
| C:ILE 167 | I:ASP 196 |
| C:THR 169 | I:VAL 198 |
| C:ARG 187 | I:LEU 199 |
| C:SER 189 | I:PRO 200 |
| C:PHE 190 | I:VAL 203 |
| C:VAL 191 | I:ALA 201 |
| C:ALA 195 | I:ARG 202 |
| C:TRP 197 |  |
| Chain D |  |
| D:TRP 171 |  |
| D:PRO 174 |  |
| D:TRP 175 |  |
| D:VAL 176 |  |
| D:ASN 180 |  |
| D:ALA 181 |  |
| D:PHE 182 |  |
| D:GLN 184 |  |
| D:PRO 185 |  |

**Interacting between Outer ring ASU and chain K ASU interacting surface area – 139.6 Å^2^**

Interacting surface residues between Outer ring ASU and chain D

| Chain D | Chain K |
| --- | --- |
| D:PRO 200 | K:ARG 167 |
| D:ALA 201 | K:GLN 205 |
| D:ARG 202 | K:TRP 208 |
| D:VAL 203 | K:PRO 210 |

**Interacting between Outer ring ASU and chain L ASU interacting surface area – 850.4 Å^2^**

| Hydrogen bonds | | Dist |
| --- | --- | --- |
| A:ASP  46[ OD1] | L:THR  89[ N  ] | 3.64 |
| A:GLU 110[ OE1] | L:ARG  39[ NH2] | 2.29 |
| A:GLY 111[ O  ] | L:GLN  37[ NE2] | 2.21 |
| A:ARG  71[ NH1] | L:THR  61[ OG1] | 3.49 |
| A:ARG  71[ NH1] | L:GLU  93[ OE1] | 3.31 |
| A:GLY 111[ N  ] | L:THR  89[ OG1] | 3.24 |
| Salt bridges | |  |
| A:ASP  46[ OD1] | L:ARG  86[ NH1] | 3.98 |
| A:GLU 110[ OE1] | L:ARG  39[ NH1] | 3.71 |
| A:GLU 110[ OE1] | L:ARG  39[ NH2] | 2.29 |
| A:GLU 110[ OE2] | L:ARG  39[ NH2] | 3.95 |
| A:ARG  71[ NH1] | L:GLU  93[ OE1] | 3.31 |
| A:ARG  71[ NH1] | L:GLU  93[ OE2] | 3.87 |

Interacting surface residues between Outer ring ASU and chain L

| Chain A | Chain L |
| --- | --- |
| A:ALA  24 | L:SER  31 |
| A:GLN  25 | L:LEU  32 |
| A:SER  26 | L:PRO  33 |
| A:PRO  27 | L:GLN  34 |
| A:THR  45 | L:GLY  35 |
| A:ASP  46 | L:GLY  36 |
| A:PRO  47 | L:GLN  37 |
| A:ASN  48 | L:PHE  38 |
| A:MET  49 | L:ARG  39 |
| A:PHE  51 | L:THR  58 |
| A:ARG  71 | L:ALA  59 |
| A:THR  73 | L:ILE  60 |
| A:THR  74 | L:THR  61 |
| A:ALA  75 | L:PRO  63 |
| A:LEU  79 | L:GLY  64 |
| A:THR  81 | L:ARG  86 |
| A:GLU 110 | L:THR  87 |
| A:GLY 111 | L:PHE  88 |
| A:ARG 112 | L:THR  89 |
| A:VAL 113 | L:PHE  91 |
| A:ARG 115 | L:GLU  93 |
| A:LYS 178 | L:THR  94 |
| Chain D | L:GLY  97 |
| D:VAL 158 | L:THR  99 |
| D:HIS 159 | L:PHE 100 |
| D:ASP 196 | L:SER 101 |
|  | L:VAL 103 |
|  | L:GLU 120 |
|  | L:LYS 129 |
|  | L:GLN 222 |
|  | D:VAL 158 |
|  | D:HIS 159 |
|  | D:ASP 196 |

**Interacting between Outer ring ASU and chain M ASU interacting surface area – 1210.4 Å^2^**

| Hydrogen bonds | | Dist |
| --- | --- | --- |
| D:ARG 164[ NH1] | M:ARG 162[ O  ] | 2.87 |
| D:ARG 162[ NH1] | M:ASP 165[ OD2] | 3.31 |
| C:PRO 174[ O  ] | M:VAL  75[ N  ] | 3.78 |
| C:ASN 180[ OD1] | M:TRP 197[ NE1] | 2.80 |
| C:VAL 176[ N  ] | M:VAL  73[ O  ] | 3.38 |
| B:GLN 184[ NE2] | M:SER  71[ OG ] | 3.02 |
| B:ARG 213[ NH1] | M:ALA  72[ O  ] | 3.89 |
| B:TYR 136[ OH ] | M:GLU  74[ OE2] | 3.12 |
| B:ARG 213[ NH1] | M:GLU  74[ OE2] | 2.62 |
| A:GLN 222[ OE1] | M:HIS 159[ NE2] | 2.48 |
| Salt bridges | |  |
| D:ARG 162[ NH1] | M:ASP 165[ OD2] | 3.31 |
| B:ARG 213[ NH2] | M:GLU  74[ OE1] | 3.46 |
| B:ARG 213[ NH1] | M:GLU  74[ OE1] | 3.54 |
| B:ARG 213[ NE ] | M:GLU  74[ OE1] | 3.45 |
| B:ARG 213[ NH2] | M:GLU  74[ OE2] | 3.92 |
| B:ARG 213[ NH1] | M:GLU  74[ OE2] | 2.62 |
| B:ARG 213[ NE ] | M:GLU  74[ OE2] | 3.15 |

Interacting surface residues between Outer ring ASU and chain M

| Chain A | Chain M |
| --- | --- |
| A:GLU 204 | M:HIS 159 |
| A:GLN 222 | M:PRO 160 |
| Chain B | M:GLN 161 |
| B:TYR 136 | M:ARG 162 |
| B:ARG 167 | M:SER 163 |
| B:GLN 184 | M:ARG 164 |
| B:LEU 185 | M:ASP 165 |
| Chain C | M:ILE 167 |
| C:ALA 173 | M:THR 169 |
| C:PRO 174 | M:ARG 187 |
| C:TRP 175 | M:SER 189 |
| C:VAL 176 | M:PHE 190 |
| C:ASP 177 | M:VAL 191 |
| C:SER 178 | M:ALA 195 |
| C:ASN 180 | M:TRP 197 |
| C:ALA 181 | M:ALA  72 |
| C:PHE 182 | M:VAL  73 |
| C:GLN 184 | M:GLU  74 |
| Chain D | M:VAL  75 |
| D:VAL 158 | M:SER  77 |
| D:HIS 159 | M:VAL 158 |
| D:PRO 160 | M:LEU 199 |
| D:GLN 161 | M:THR  70 |
| D:ARG 162 | M:SER  71 |
| D:SER 163 | M:PRO  76 |
| D:ARG 164 | M:ALA  78 |
| D:ASP 165 | M:SER  79 |
| D:ILE 167 | M:THR 157 |
| D:THR 169 |  |
| D:ARG 187 |  |
| D:SER 189 |  |
| D:PHE 190 |  |
| D:VAL 191 |  |
| D:ALA 195 |  |
| D:TRP 197 |  |

**Interacting between Outer ring ASU and chain N ASU interacting surface area – 334.4 Å^2^**

Interacting surface residues between Outer ring ASU and chain N

| Chain D | Chain N |
| --- | --- |
| D:VAL 158 | N:TRP 171 |
| D:HIS 159 | N:PRO 174 |
| D:PRO 160 | N:TRP 175 |
| D:ARG 162 | N:VAL 176 |
| D:ALA 195 | N:ASN 180 |
| D:ASP 196 | N:ALA 181 |
| D:TRP 197 | N:PHE 182 |
| D:VAL 198 | N:GLN 184 |
| D:LEU 199 | N:PRO 185 |
| D:PRO 200 |  |
| D:VAL 203 |  |

**Interacting between Outer ring ASU and chain O ASU interacting surface area – 224.6 Å^2^**

| Hydrogen bonds | | Dist |
| --- | --- | --- |
| O:MET 180[ N  ] | A:ASP  69[ OD2] | 3.10 |

Interacting surface residues between Outer ring ASU and chain O

| Chain A | Chain O |
| --- | --- |
| A:PRO  47 | O:PRO 177 |
| A:ALA  68 | O:GLY 178 |
| A:ASP  69 | O:MET 179 |
| A:LYS  70 | O:MET 180 |
| A:ARG  71 | O:SER 182 |
| A:LEU  79 | O:GLN 183 |
| A:PHE  80 | O:GLU 184 |
| A:THR  81 |  |
| A:VAL  83 |  |
