# Supplementary material for: Architecture of the outer-membrane core complex from a conjugative type IV secretion system
Source: Nat Commun. 2021 Nov 25;12:6834. doi: 10.1038/s41467-021-27178-8 (PMC8617172; doi:10.1038/s41467-021-27178-8)
Supplement: Supplementary file 2 — Reporting Summary [file 41467_2021_27178_MOESM2_ESM.pdf]

nature portfolio

Corresponding author(s): Tiago R D Costa
Aravindan Ilangovan

Last updated by author(s): Oct 21, 2021

Reporting Summary

Nature Portfolio wishes to improve the reproducibility of the work that we publish. This form provides structure for consistency and transparency in reporting. For further information on Nature Portfolio policies, see our Editorial Policies and the Editorial Policy Checklist.

Statistics

For all statistical analyses, confirm that the following items are present in the figure legend, table legend, main text, or Methods section.

n/a	Confirmed	
☐	☒	The exact sample size (*n*) for each experimental group/condition, given as a discrete number and unit of measurement
☐	☒	A statement on whether measurements were taken from distinct samples or whether the same sample was measured repeatedly
☒	☐	The statistical test(s) used AND whether they are one- or two-sided<br>*Only common tests should be described solely by name; describe more complex techniques in the Methods section.*
☒	☐	A description of all covariates tested
☒	☐	A description of any assumptions or corrections, such as tests of normality and adjustment for multiple comparisons
☒	☐	A full description of the statistical parameters including central tendency (e.g. means) or other basic estimates (e.g. regression coefficient) AND variation (e.g. standard deviation) or associated estimates of uncertainty (e.g. confidence intervals)
☒	☐	For null hypothesis testing, the test statistic (e.g. *F*, *t*, *r*) with confidence intervals, effect sizes, degrees of freedom and *P* value noted<br>*Give P values as exact values whenever suitable.*
☒	☐	For Bayesian analysis, information on the choice of priors and Markov chain Monte Carlo settings
☒	☐	For hierarchical and complex designs, identification of the appropriate level for tests and full reporting of outcomes
☒	☐	Estimates of effect sizes (e.g. Cohen's *d*, Pearson's *r*), indicating how they were calculated

Our web collection on statistics for biologists contains articles on many of the points above.

Software and code

Policy information about availability of computer code

Data collection	Thermo Fisher Scientific EPU 2
Data analysis	CryoSparc v.3.1.0; Relion 3.1 software; Phenix package v1.18.2 (Real-Space-Refine tool;Comprehensive validation tool); Coot v0.9.4.1; Chimera v1.12; ChimeraX v1.2; PyMol v2.4.1

For manuscripts utilizing custom algorithms or software that are central to the research but not yet described in published literature, software must be made available to editors and reviewers. We strongly encourage code deposition in a community repository (e.g. GitHub). See the Nature Portfolio guidelines for submitting code & software for further information.

Data

Policy information about availability of data

All manuscripts must include a data availability statement. This statement should provide the following information, where applicable:

- Accession codes, unique identifiers, or web links for publicly available datasets
- A description of any restrictions on data availability
- For clinical datasets or third party data, please ensure that the statement adheres to our policy

The atomic coordinates have been deposited at the Protein Data Bank with accession codes 7OKN (OMCC IR) and 7OKO (OMCC OR). The density maps have been deposited at the Electron Microscopy Data Bank with accession codes EMD-12962 (OMCC IR), EMD-12963 (OMCC OR) and EMD-13231 (OMCC C1).
Maps and Models will be available upon publication.

Field-specific reporting

Please select the one below that is the best fit for your research. If you are not sure, read the appropriate sections before making your selection.

☒ Life sciences ☐ Behavioural & social sciences ☐ Ecological, evolutionary & environmental sciences

For a reference copy of the document with all sections, see nature.com/documents/nr-reporting-summary-flat.pdf

Life sciences study design

All studies must disclose on these points even when the disclosure is negative.

Sample size	A total of 11588 cryo-electron microscopy movies were collected from the OMCC sample, and an initial set of 1445958 particles were processed (see Table 1 for details). Sample sizes were those required for the resolution.
Data exclusions	Final map was obtained upon standard 2D and 3D classification approaches were used in CryoSparc to exclude data.
Replication	Two randomly divided half datasets were processed independently, and combined to obtain the final 3D map. Resolution was accessed by comparing two independent mmaps.
Randomization	Data collection was randomly set by the EPU software. CryoEM particles were randomly divided into two groups as a standard approach.
Blinding	cryoEM dataset was blindly collected.

Reporting for specific materials, systems and methods

We require information from authors about some types of materials, experimental systems and methods used in many studies. Here, indicate whether each material, system or method listed is relevant to your study. If you are not sure if a list item applies to your research, read the appropriate section before selecting a response.

Materials & experimental systems

n/a	Involved in the study	
☒	☐	Antibodies
☒	☐	Eukaryotic cell lines
☒	☐	Palaeontology and archaeology
☒	☐	Animals and other organisms
☒	☐	Human research participants
☒	☐	Clinical data
☒	☐	Dual use research of concern

Methods

n/a	Involved in the study	
☒	☐	ChIP-seq
☒	☐	Flow cytometry
☒	☐	MRI-based neuroimaging
